# Genome-Wide Association of Body Fat Distribution in African Ancestry Populations Suggests New Loci

**DOI:** 10.1371/journal.pgen.1003681

**Published:** 2013-08-15

**Authors:** Ching-Ti Liu, Keri L. Monda, Kira C. Taylor, Leslie Lange, Ellen W. Demerath, Walter Palmas, Mary K. Wojczynski, Jaclyn C. Ellis, Mara Z. Vitolins, Simin Liu, George J. Papanicolaou, Marguerite R. Irvin, Luting Xue, Paula J. Griffin, Michael A. Nalls, Adebowale Adeyemo, Jiankang Liu, Guo Li, Edward A. Ruiz-Narvaez, Wei-Min Chen, Fang Chen, Brian E. Henderson, Robert C. Millikan, Christine B. Ambrosone, Sara S. Strom, Xiuqing Guo, Jeanette S. Andrews, Yan V. Sun, Thomas H. Mosley, Lisa R. Yanek, Daniel Shriner, Talin Haritunians, Jerome I. Rotter, Elizabeth K. Speliotes, Megan Smith, Lynn Rosenberg, Josyf Mychaleckyj, Uma Nayak, Ida Spruill, W. Timothy Garvey, Curtis Pettaway, Sarah Nyante, Elisa V. Bandera, Angela F. Britton, Alan B. Zonderman, Laura J. Rasmussen-Torvik, Yii-Der Ida Chen, Jingzhong Ding, Kurt Lohman, Stephen B. Kritchevsky, Wei Zhao, Patricia A. Peyser, Sharon L. R. Kardia, Edmond Kabagambe, Ulrich Broeckel, Guanjie Chen, Jie Zhou, Sylvia Wassertheil-Smoller, Marian L. Neuhouser, Evadnie Rampersaud, Bruce Psaty, Charles Kooperberg, JoAnn E. Manson, Lewis H. Kuller, Heather M. Ochs-Balcom, Karen C. Johnson, Lara Sucheston, Jose M. Ordovas, Julie R. Palmer, Christopher A. Haiman, Barbara McKnight, Barbara V. Howard, Diane M. Becker, Lawrence F. Bielak, Yongmei Liu, Matthew A. Allison, Struan F. A. Grant, Gregory L. Burke, Sanjay R. Patel, Pamela J. Schreiner, Ingrid B. Borecki, Michele K. Evans, Herman Taylor, Michele M. Sale, Virginia Howard, Christopher S. Carlson, Charles N. Rotimi, Mary Cushman, Tamara B. Harris, Alexander P. Reiner, L. Adrienne Cupples, Kari E. North, Caroline S. Fox

**Affiliations:** 1 Department of Biostatistics, Boston University School of Public Health, Boston, Massachusetts, United States of America; 2 Department of Epidemiology, University of North Carolina at Chapel Hill, Chapel Hill, North Carolina, United States of America; 3 Department of Epidemiology and Population Health, University of Louisville, Louisville, Kentucky, United States of America; 4 Department of Genetics, UNC School of Medicine, Chapel Hill, North Carolina, United States of America; 5 University of Minnesota, Minneapolis, Minnesota, United States of America; 6 Department of Medicine, Columbia University, New York, New York, United States of America; 7 Department of Genetics, Washington University School of Medicine, St. Louis, Missouri, United States of America; 8 Department of Epidemiology & Prevention, Wake Forest University Health Sciences, Winston-Salem, North Carolina, United States of America; 9 Departments of Epidemiology, Medicine, and Obstetrics and Gynecology and Center for Metabolic Disease Prevention, Los Angeles, California, United States of America; 10 Division of Cardiovascular Sciences, Prevention and Population Sciences Program, National Heart, Lung, & Blood Institute, Bethesda, Maryland, United States of America; 11 Department of Epidemiology, UAB, Birmingham, Alabama, United States of America; 12 Laboratory of Neurogenetics, National Institute of Aging, National Institutes of Health, Bethesda, Maryland, United States of America; 13 Center for Research on Genomics and Global Health, National Human Genome Research Institute, Bethesda, Maryland, United States of America; 14 Department of Medicine, University of Mississippi Medical Center, Jackson, Mississippi, United States of America; 15 University of Washington, Seattle, Washington, United States of America; 16 Slone Epidemiology Center, Boston University, Boston, Massachusetts, United States of America; 17 Center for Public Health and Genomics and Department of Public Health Sciences, University of Virginia, Charlottesville, Virginia, United States of America; 18 Department of Preventive Medicine, Keck School of Medicine, University of Southern California, Los Angeles, California, United States of America; 19 Department of Epidemiology, Gillings School of Global Public Health, and Lineberger Comprehensive Cancer Center, UNC at Chapel Hill, Chapel Hill, North Carolina, United States of America; 20 Department of Cancer Prevention and Control, Roswell Park Cancer Institute, Buffalo, New York, United States of America; 21 Department of Epidemiology, The University of Texas M. D. Anderson Cancer Center, Houston, Texas, United States of America; 22 Medical Genetics Institute, Cedars-Sinai Medical Center, Los Angeles, California, United States of America; 23 Department of Biostatistical Sciences, Wake Forest School of Medicine, Winston-Salem, North Carolina, United States of America; 24 Department of Epidemiolgy, School of Public Health, University of Michigan, Ann Arbor, Michigan, United States of America; 25 Division of Geriatrics, Department of Medicine, University of Mississippi Medical Center, Jackson, Mississippi, United States of America; 26 Department of Medicine, Johns Hopkins School of Medicine, Baltimore, Maryland, United States of America; 27 University of Michigan, Ann Arbor, Michigan, United States of America; 28 Department of Biostatistics, University of Washington, Seattle, Washington, United States of America; 29 Medical University of South Carolina, Charleston, South Carolina, United States of America; 30 Department of Epidemiology, UAB School of Public Health, Birmingham, Alabama, United States of America; 31 Department of Urology, The University of Texas M.D. Anderson Cancer Center, Houston, Texas, United States of America; 32 The Cancer Institute of New Jersey, New Brunswick, New Jersey, United States of America; 33 Laboratory of Personality and Cognition, National Institute on Aging, National Institutes of Health, NIH Biomedical Center, Baltimore, Maryland, United States of America; 34 Department of Preventive Medicine, Northwestern University Feinberg School of Medicine, Chicago, Illinois, United States of America; 35 Department of Internal Medicine/Geriatrics, Wake Forest University School of Medicine, Winston-Salem, North Carolina, United States of America; 36 Department of Epidemiology & Prevention, Public Health Sciences, Wake Forest School of Medicine, Winston-Salem, North Carolina, United States of America; 37 Department of Internal Medicine/Geriatrics, Wake Forest School of Medicine, Winston-Salem, North Carolina, United States of America; 38 Department of Epidemiolgy, School of Public Health, University of Michigan, Ann Arbor, Michigan, United States of America; 39 Department of Pediatrics, Medicine and Physiology, Medical College of Wisconsin, Milwaukee, Wisconsin, United States of America; 40 Department of Epidemiology and Population Health, Albert Einstein College of Medicine, Bronx, New York, New York, United States of America; 41 Division of Public Health Sciences, Fred Hutchinson Cancer Research Center, Seattle, Washington, United States of America; 42 Miami Institute for Human Genomics, Miami, Florida, United States of America; 43 John T. McDonald Department of Human Genetics, University of Miami, Miami, Florida, United States of America; 44 Cardiovascular Health Research Unit, Departments of Medicine, Epidemiology, and Health Services and Group Health Research Institute, Group Health Cooperative, Seattle, Washington, United States of America; 45 Division of Public Health Sciences, Fred Hutchinson Cancer Research Center, Seattle, Washington, United States of America; 46 Department of Medicine, Brigham and Women's Hospital, Harvard Medical School, Boston, Massachusetts, United States of America; 47 Department of Epidemiology, University of Pittsburgh, Graduate School of Public Health, Pittsburgh, Pennsylvania, United States of America; 48 Department of Social and Preventive Medicine, University at Buffalo, Buffalo, New York, United States of America; 49 Department of Preventive Medicine, University of Tennessee Health Science Center, Memphis, Tennessee, United States of America; 50 Department of Biostatistics, University of Buffalo School of Public Health and Health Professions, New York State Center of Excellence in Bioinformatics and Life Sciences, Buffalo, New York, United States of America; 51 Tufts University, Boston, Massachusetts, United States of America; 52 Department of Biostatistics, University of Washington, Seattle, Washington, United States of America; 53 MedStar Health Research Institute and Georgetown University, Hyattsville, Maryland, United States of America; 54 Department of Epidemiology and Prevention, Public Health Sciences, Wake Forest School of Medicine, Winston-Salem, North Carolina, United States of America; 55 University of California at San Diego Department of Preventive Medicine, La Jolla, California, United States of America; 56 Division of Human Genetics, Children's Hospital of Philadelphia Research Institute, Philadelphia, Pennsylvania, United States of America; 57 Division of Public Health Sciences, Wake Forest School of Medicine, Winston-Salem, North Carolina, United States of America; 58 Division of Sleep Medicine, Brigham and Women's Hospital, Boston, Massachusetts, United States of America; 59 Division of Epidemiology and Community Health, University of Minnesota, Minneapolis, Minnesota, United States of America; 60 Health Disparities Research Section, Clinical Research Branch, National Institute on Aging, National Institutes of Health, NIH Biomedical Center, Baltimore, Maryland, United States of America; 61 Center for Public Genomics, Department of Biochemistry and Molecular Genetics and Department of Medicine, University of Virginia, Charlottesville, Virginia, United States of America; 62 Department of Medicine, University of Vermont, Colchester, Vermont, United States of America; 63 Laboratory of Epidemiology, Demography, and Biometry, NIA, Bethesda, Maryland, United States of America; 64 Department of Epidemiology, University of Washington, Seattle, Washington, United States of America; 65 NHLBI's Framingham Heart Study, Framingham, Massachusetts, United States of America; 66 NHLBI's Center for Population Studies, Framingham, Massachusetts, United States of America; 67 Division of Endocrinology, Brigham and Women's Hospital and Harvard Medical School, Boston, Massachusetts, United States of America; University of Oxford, United Kingdom

## Abstract

Central obesity, measured by waist circumference (WC) or waist-hip ratio (WHR), is a marker of body fat distribution. Although obesity disproportionately affects minority populations, few studies have conducted genome-wide association study (GWAS) of fat distribution among those of predominantly African ancestry (AA). We performed GWAS of WC and WHR, adjusted and unadjusted for BMI, in up to 33,591 and 27,350 AA individuals, respectively. We identified loci associated with fat distribution in AA individuals using meta-analyses of GWA results for WC and WHR (stage 1). Overall, 25 SNPs with single genomic control (GC)-corrected p-values<5.0×10^−6^ were followed-up (stage 2) in AA with WC and with WHR. Additionally, we interrogated genomic regions of previously identified European ancestry (EA) WHR loci among AA. In joint analysis of association results including both Stage 1 and 2 cohorts, 2 SNPs demonstrated association, rs2075064 at *LHX2*, *p = *2.24×10^−8^ for WC-adjusted-for-BMI, and rs6931262 at *RREB1*, *p = *2.48×10^−8^ for WHR-adjusted-for-BMI. However, neither signal was genome-wide significant after double GC-correction (*LHX2*: p = 6.5×10^−8^; *RREB1*: p = 5.7×10^−8^). Six of fourteen previously reported loci for waist in EA populations were significant (p<0.05 divided by the number of independent SNPs within the region) in AA studied here (*TBX15-WARS2*, *GRB14*, *ADAMTS9*, *LY86*, *RSPO3*, *ITPR2-SSPN*). Further, we observed associations with metabolic traits: rs13389219 at *GRB14* associated with HDL-cholesterol, triglycerides, and fasting insulin, and rs13060013 at *ADAMTS9* with HDL-cholesterol and fasting insulin. Finally, we observed nominal evidence for sexual dimorphism, with stronger results in AA women at the *GRB14* locus (p for interaction = 0.02). In conclusion, we identified two suggestive loci associated with fat distribution in AA populations in addition to confirming 6 loci previously identified in populations of EA. These findings reinforce the concept that there are fat distribution loci that are independent of generalized adiposity.

## Author Summary

Central obesity is a marker of body fat distribution and is known to have a genetic underpinning. Few studies have reported genome-wide association study (GWAS) results among individuals of predominantly African ancestry (AA). We performed a collaborative meta-analysis in order to identify genetic loci associated with body fat distribution in AA individuals using waist circumference (WC) and waist to hip ratio (WHR) as measures of fat distribution, with and without adjustment for body mass index (BMI). We uncovered 2 genetic loci potentially associated with fat distribution: *LHX2* in association with WC-adjusted-for-BMI and at *RREB1* for WHR-adjusted-for-BMI. Six of fourteen previously reported loci for waist in EA populations were significant in AA studied here (*TBX15-WARS2*, *GRB14*, *ADAMTS9*, *LY86*, *RSPO3*, *ITPR2-SSPN*). These findings reinforce the concept that there are loci for body fat distribution that are independent of generalized adiposity.

## Introduction

Obesity is an important public health problem, reaching epidemic proportions. The prevalence varies by ethnicity, with nearly one-third of European ancestry (EA) and almost one-half of African ancestry (AA) Americans considered obese [1]. Recent studies have suggested that body fat distribution, above and beyond generalized adiposity, is an important metric of metabolic health, as different fat compartments are associated with differential metabolic risk [2]. Specifically, a tendency to deposit fat centrally is associated with diabetes, hypertension, and heart disease [3]–[6], even after accounting for generalized adiposity [7], [8].

Waist circumference (WC) and waist to hip ratio (WHR) are established measures of body fat distribution [9] that differ by ethnicity [10], [11] and demonstrate a genetic component. Twin studies documented heritability of levels for WC and WHR in EA and AA individuals, ranging from 31%–76% [12]–[15] even after accounting for BMI [14]–[16]. A recent meta-analysis of WHR in EA individuals identified 14 loci for body fat distribution [17]. In addition, recent studies among EAs for percent body fat, fatty liver, visceral fat, and pericardial fat reported unique loci for fat distribution and ectopic fat depots above and beyond those associated with generalized adiposity [18]–[21]. Similar studies are not available in AA populations. Thus, the purpose of the present analysis was to perform a collaborative large-scale meta-analysis of waist-based traits in AA individuals.

## Results

We analyzed genetic loci for waist circumference (WC) and waist hip ratio (WHR) in up to 23,564 AA participants in the discovery set (Stage 1) and 10,027 AA participants in the replication set (Stage 2). **Table 1** presents overall study sample characteristics and **Supplementary Table S1** presents stratified sample characteristics by gender. Detailed descriptions of each cohort are shown in **Supplementary Table S2** and the **Supplementary Materials Text S1**. In our GWAS analysis, we applied single genomic control (GC) correction to avoid the overly conservative double GC correction [22], [23] but we also provide double GC-corrected p-values for the joint meta-analysis of stage 1 and stage 2 samples (**Table 2**).

**Table 1:** Study sample characteristics.

Study	Sample Size WC/Hip	European Ancestry %	Women %	Age (years)	WC (cm)	Hip (cm)	WHR	BMI
	n/n	median, Q1/Q3	% (n)	mean (SD)	mean (SD)	mean (SD)	mean (SD)	mean (SD)
**Stage 1 Cohorts**								
WHI-SHARe	8138/8128	NA	100 (8155)	61.6 (7.0)	91.5 (13.4)	111.2 (12.7)	0.82 (0.07)	31.0 (6.4)
HANDLS	961/961	0.16,0.11/0.22	55.4 (532)	48.5 (9.0)	98.5 (17.5)	107.5 (16.4)	0.91 (0.07)	29.9 (8.0)
MESA/SHARe (Family)	946/946	0.22, 0.12/0.30	60 (573)	58.2 (8.7)	100.6 (16.2)	110.1 (13.2)	0.91 (0.08)	30.7 (6.4)
Health ABC	1137/0	0.22, 0.12/0.33	57.2 (650)	73.4 (2.9)	100.3 (13.8)	NA	NA	28.6 (5.4)
GENOA	996/996	0.14, 0.09/0.21	70.4(701)	56.4 (11.2)	102.7 (17.1)	113.3 (15.0)	0.91 (0.08)	31.1 (6.8)
GeneSTAR	1081/552	0.13, 0.09/0.19	61.7 (667)	42.8 (10.4)	98.7 (17.0)	111.5 (14.6)	0.88 (0.08)	31.1 (7.3)
Family Heart Study	624/624	0.13, 0.09/0.19	65.7 (410)	53.3 (10.8)	104.1 (16.8)	114.1 (15.7)	0.92 (0.07)	32.7 (7.4)
HyperGEN	1179/1168	0.15, 0.11/0.20	66 (778)	45.1 (13.0)	102.9 (18.5)	114.4 (16.1)	0.89 (0.08)	32.4 (7.9)
HUFS	924/924	0.20, 0.14/0.27	57.9 (535)	45.5 (12.1)	95.0 (16.7)	110.2 (16.1)	0.86 (0.08)	30.2 (8.2)
CARe Studies*								
ARIC	2778/2778	0.15, 0.11/0.22	63.2 (1755)	53.3 (5.8)	99.2 (15.0)	107.8 (11.9)	0.92 (0.08)	30.0 (6.0)
CARDIA	819/818	0.17, 0.12/0.23	60.9 (499)	25.4 (3.2)	79.1 (12.3)	102.1 (12.2)	0.77 (0.07)	25.7 (5.8)
CFS	468/468	0.18, 0.13/0.25	59.4 (278)	44.6 (15.2)	102.3 (20.2)	115.9 (18.3)	0.88 (0.09)	34.2 (9.5)
JHS	2132/0	0.16, 0.12/0.21	60.7 (1295)	50.0 (12.1)	101.2 (17.0)	NA	NA	32.3 (7.8)
MESA	1381/1381	0.19, 0.12/0.30	54.5 (753)	61.6 (9.9)	101.2 (14.5)	109.8 (11.8)	0.92 (0.08)	30.2 (5.7)
**Stage 2 Cohorts**								
CHS	819/820	0.24, 0.16/0.36	63 (517)	72.9 (5.7)	98.8 (14.4)	104.6 (12.0)	0.94 (0.07)	28.5 (5.5)
BWHS	1499/1499	0.18, 0.11/0.25	100 (1499)	46.9 (10.1)	82.5 (13.0)	105.4 (12.9)	0.78 (0.09)	28.0 (5.7)
REGARDS_Diabetes_CASES	1136/0	0.13, 0.07/0.21	64(730)	63.4(8.7)	104.1(14.5)	NA	NA	30.2(7.0)
REGARDS_CONTROLS	1244/0	0.14, 0.08/0.22	64(796)	63.1(8.5)	104.2(14.3)	NA	NA	30.1(7.2)
SUGAR_Diabetes_CASES	865/855	0.05, 0.02/0.09	78(759)	54.1(14)	99.7 (16.7)	108.4(12.5)	0.92(0.08)	34.1(7.9)
SUGAR_CONTROLS	182/181	0.05, 0.02/0.11	72(144)	54.2(14.1)	99.5 (16.8)	114.3(12.6)	0.87(0.09)	34(7.6)
MEC Breast Cancer Cases	259/258	NA	100 (259)	71.6(8.6)	96.0(14.3)	109.1(14.0)	0.88(0.10)	28.6(5.8)
MEC Breast Cancer Controls	528/527	NA	100 (528)	70.9(8.4)	95.4(14.8)	110.2(14.3)	0.87(0.09)	28.7(6.0)
CBCS Breast Cancer Cases	626/626	NA	100 (626)	51.3(11.9)	96.6(14.2)	113.3(13.8)	0.85(0.08)	31.9(7.2)
CBCS Breast Cancer Controls	579/579	NA	100 (579)	51.8(11.4)	95.5(15.1)	114.3(13.9)	0.84(0.08)	32.3(7.5)
WCHS Breast Cancer Cases	256/259	NA	100 (259)	49.8(9.7)	101.1(16.7)	114.0(13.9)	0.88(0.08)	31.9(7.1)
WCHS Breast Cancer Controls	237/237	NA	100 (237)	49.8(9.4)	98.7(15.6)	113.0(13.7)	0.87(0.07)	31.3(7.0)
MEC Prostate Cancer Cases	542/528	NA	0 (0)	73.8(7.0)	99.5(12.4)	105.5(10.9)	0.94(0.09)	27.1(4.0)
MEC Prostate Cancer Controls	877/856	NA	0 (0)	69.6(8.5)	99.4(12.7)	105.8(12.0)	0.94(0.11)	27.3(3.8)
MDA Prostate Cancer Cases	153/153	NA	0 (0)	59.2(7.8)	100.5(13.0)	107.9(11.3)	0.93(0.06)	27.1(5.1)
MDA Prostate Cancer Controls	228/228	NA	0 (0)	59.6(8.7)	99.7(12.2)	106.9(11.2)	0.93(0.06)	29.1(5.0)

**Table 2:** SNPs associated with waist-related trait at p<5.0E-6 in Stage 1.

							Stage 1			Stage 2			Combined					
Trait	SNP	chr	bp (b36)	Gene	All^1^	EAF^2^	beta	SE	P-val	beta	SE	P-val^3^	beta	SE	P-val	*P_2GC_* 4
WC_BMI_pooled	rs2075064	9	125823668	*LHX2*	t/c	0.13	−0.08	0.01	5.5E-08	−0.04	0.02	3.2E-02	−0.07	0.01	2.2E-08	6.5E-08
WHR_BMI_pooled	rs6931262	6	7162516	*RREB1*	t/c	0.25	0.07	0.01	5.3E-08	0.04	0.02	4.5E-02	0.06	0.01	2.5E-08	5.7E-08
WC_men	rs6867983	5	55889910	*MAP3K1*	t/c	0.24	−0.11	0.02	3.5E-07	−0.06	0.03	2.5E-02	−0.09	0.02	1.4E-07	2.7E-07
WC_pooled	rs7601155	2	28201186	*BRE*	t/c	0.13	0.07	0.01	2.9E-07	0.04	0.02	4.6E-02	0.06	0.01	1.7E-07	4.9E-07
WHR_BMI_pooled	rs10894604	11	132146956	*OPCML*	t/g	0.83	−0.07	0.01	1.3E-06	−0.04	0.03	4.1E-02	−0.06	0.01	3.8E-07	7.7E-07
WHR_BMI_Men	rs17213965	16	15789468	*MYH11*	t/c	0.14	0.17	0.03	3.9E-08	0.03	0.04	2.2E-01	0.12	0.02	8.8E-07	1.3E-06
WC_Men	rs2570467	5	95882435	*PCSK1*	a/g	0.86	−0.14	0.03	3.8E-08	−0.02	0.04	2.6E-01	−0.10	0.02	1.2E-06	2.1E-06
WHR_BMI_men	rs11777345	8	5413939	*CSMD1*	c/g	0.90	0.24	0.04	1.1E-07	−0.06	0.10	7.1E-01	0.19	0.04	3.2E-06	4.8E-06
WC_BMI_men	rs4730779	7	116810771	*ASZ1*	a/g	0.86	0.14	0.03	1.9E-07	0.03	0.04	2.6E-01	0.10	0.02	3.8E-06	6.3E-06
WHR_BMI_women	rs6739392	2	67455475	*ETAA1*	a/t	0.23	0.07	0.01	7.4E-07	−0.02	0.05	6.7E-01	0.06	0.01	4.0E-06	6.2E-06
WC_Men	rs1345301	2	102242019	*IL1RL2; IL1RL1*	a/g	0.75	0.12	0.02	4.0E-08	0.01	0.03	3.6E-01	0.08	0.02	4.6E-06	7.9E-06
WC_women	rs16830366	2	199293801	*SATB2*	t/c	0.07	0.11	0.02	4.5E-07	0.01	0.04	3.6E-01	0.09	0.02	6.2E-06	1.0E-05
WC_women	rs11961438	6	154688273	*PIP3-E*	a/g	0.22	0.07	0.01	9.6E-07	0.01	0.02	3.5E-01	0.05	0.01	9.7E-06	1.5E-05
WC_pooled	rs7672174	4	155860227	*LRAT*	a/g	0.80	0.06	0.01	3.2E-07	0.00	0.02	5.0E-01	0.04	0.01	1.2E-05	2.7E-05
WHR_pooled	rs4292018	18	18956127	*CABLES1*	c/g	0.59	0.06	0.01	7.9E-07	0.00	0.02	4.7E-01	0.04	0.01	1.7E-05	2.6E-05
WC_pooled	rs1355187	4	131562229	*C4orf33*	a/g	0.37	−0.05	0.01	4.8E-07	0.00	0.02	5.3E-01	−0.04	0.01	2.2E-05	4.2E-05
WC_men	rs2907512	12	3922018	*PARP11*	a/c	0.94	−0.27	0.05	9.1E-07	−0.05	0.06	1.9E-01	−0.16	0.04	3.0E-05	4.5E-05
WC_BMI_pooled	rs12777819	10	87883768	*GRID1*	t/c	0.26	−0.06	0.01	2.8E-07	0.01	0.02	6.7E-01	−0.04	0.01	3.3E-05	5.4E-05
WHR_men	rs11197515	10	117807673	*GFRA1*	t/c	0.19	−0.13	0.03	1.7E-06	−0.01	0.04	3.9E-01	−0.09	0.02	4.8E-05	6.9E-05
WHR_pooled	rs214679	12	45963655	*FAM113B*	t/g	0.08	0.10	0.02	8.2E-07	−0.02	0.03	7.5E-01	0.07	0.02	9.4E-05	1.4E-04
WHR_pooled	rs17134146	7	4006655	*SDK1*	c/g	0.78	0.06	0.01	1.8E-07	−0.03	0.02	9.3E-01	0.04	0.01	1.3E-04	1.8E-04
WC_BMI_pooled	rs2881189	7	5891682	*OCM*	a/g	0.84	−0.08	0.02	4.3E-07	0.03	0.02	8.8E-01	−0.04	0.01	6.1E-04	8.9E-04
WC_BMI_men	rs2548774	5	65084635	*NLN*	t/c	0.28	0.11	0.02	9.4E-07	−0.03	0.03	8.7E-01	0.06	0.02	1.4E-03	1.8E-03
WC_pooled	rs2958759	8	30247097	*DCTN6*	a/c	0.67	−0.05	0.01	8.9E-07	0.04	0.02	9.9E-01	−0.03	0.01	2.6E-03	3.7E-03
WHR_men	rs7296740	12	2966159	*TEAD4*	t/c	0.68	0.11	0.02	3.7E-06	−0.05	0.03	9.4E-01	0.06	0.02	3.4E-03	4.2E-03

1 effect allele/other allele.

2 effect allele frequency.

3 one-side test p-value.

4 P_2GC_: double GC-corrected p-value.

### Stage 1 Genome-Wide Association Analyses for WC and WHR

We conducted genome-wide association analyses for 3.2 million variants, including genotyped and imputed variants, among AA individuals for WC, WC adjusted for BMI (WC-BMI), WHR and WHR adjusted for BMI (WHR-BMI) within each cohort, overall and by sex, and meta-analyzed the results. The Quantile-Quantile and Manhattan plots for all analyses are displayed in **Supplementary Figure S1**. With concern of overly conservative double GC-correction, we applied single GC-correction p values to select variants for follow-up. Three loci had p<5×10^−8^ under single GC-correction; rs2570467 at *PCSK1* with WC, rs1345301 at *IL1RL2* with WC, and rs17213965 at *MYH11* with WHR-BMI, all in men (n = 5967, 5973, 4398, respectively). Across all traits analyzed, an additional 22 independent SNPs had a single GC-corrected p<5.0×10^−6^ (**Table 2**). Heterogeneity tests were examined across all cohorts and none of these 25 SNPs were significant (all p-values>0.05/25) in heterogeneity testing after adjusting for multiple testing, indicating that we did not observe statistically different allelic effects for these 25 SNPs across the participating studies.

### Stage 2 Analyses and Joint Meta-Analysis of Stage 1 and Stage 2

**Figure 1:**
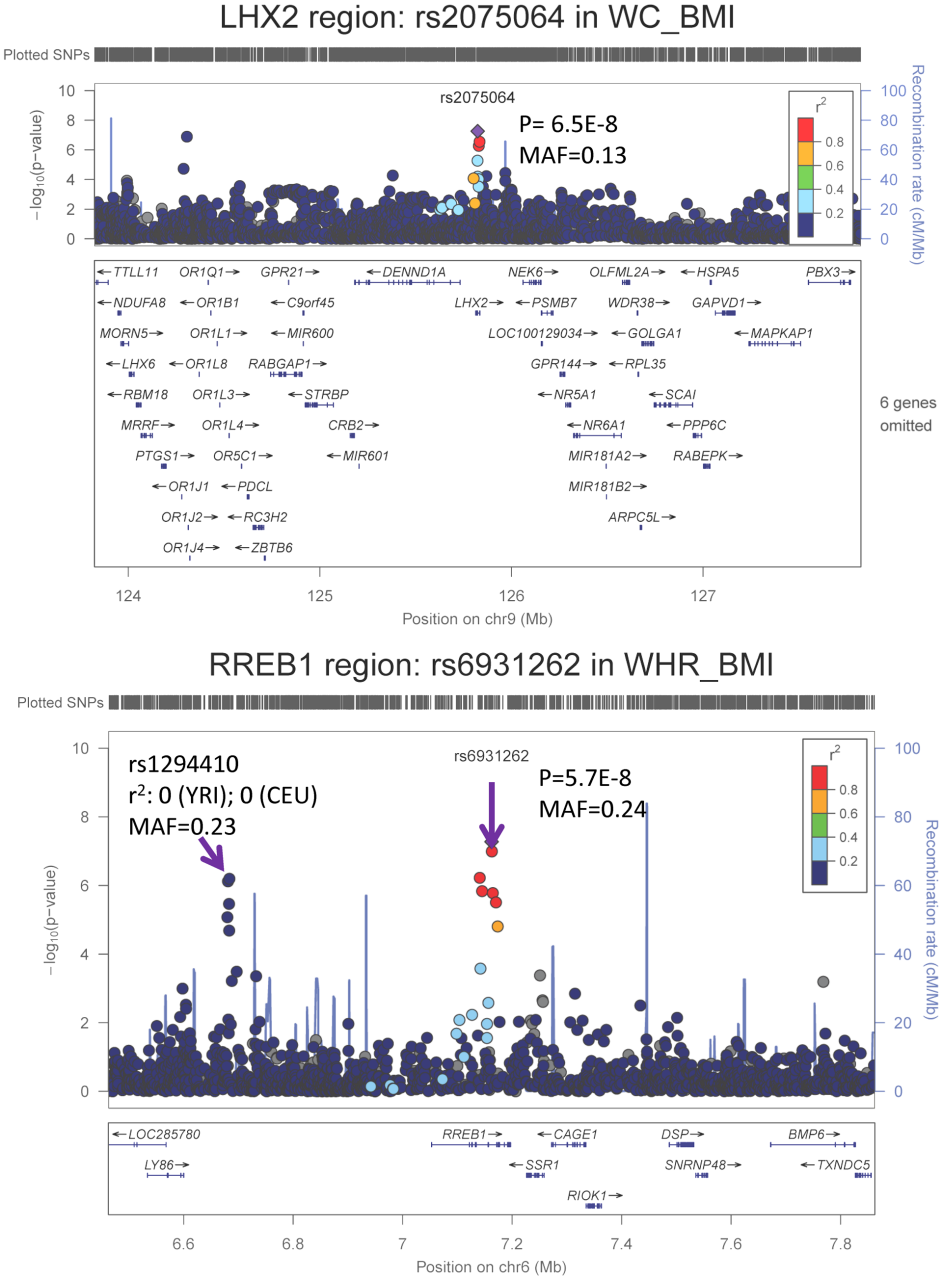
Regional association plots based on single GC-corrected p-value for *LHX2* and *RREB1*, Stage 1 only. MAF = minor allele frequency. The p-values for the index SNP rs2075064 in *LHX2* loci are 5.5E-8, 0.03, and 2.2E-8 for Stage 1, Stage 2 and joint analysis. The p-values for the index SNP rs6931262 at *RREB1* loci are 5.3E-8, 0.02 and 2.5E-8 for Stage 1, Stage 2 and joint analysis. The double GC-corrected p-value for the joint analysis are 6.5E-8, 5.7E-8 and 1.8E-6 for rs2075064, rs6931262 and rs1294410, respectively.

We carried forward all 25 SNPs with single GC-corrected p<5×10^−6^ from stage 1 and tested their association in Stage 2 with the traits of interest either in the gender-specific or gender-combined data depending on the findings in Stage 1, in up to 10,027 AA individuals with WC and 7,606 AA individuals with WHR. Significance was defined as the joint meta-analysis of stage 1 and stage 2 p-value<5×10^−8^. Results for these SNPs from discovery, validation and joint analyses are shown in **Table 2**, and the imputation quality for these SNPs is provided in **Supplementary Table S3**. Three SNPs with p<5×10^−8^ in the men only analysis of Stage 1 failed to replicate (p>5×10^−8^, n<6,000 in stage 1 and n<3,250 in stage 2) but two of the 25 SNPs carried forward from Stage 1 reached genome-wide significance under single genomic control (GC) in the joint meta-analysis of Stage 1 and Stage 2 data: rs2075064 (*LHX2*, *p = *2.24×10^−8^) in association with WC-BMI, and rs6931262 (*RREB1*, *p = *2.48×10^−8^) in association with WHR-BMI. We note, however, that double GC-corrected p values for these two variants have slightly attenuated p-values: rs2075064 (*LHX2*, p = 6.5×10^−8^) and rs6931262 (*RREB1*, p = 5.7×10^−8^), which were no longer genome-wide significant. The regional association plots for these two loci are presented in **Figure 1**. The lead SNP rs6931262 at *RREB1* is 474 kb away from rs1294421 at *LY86*, previously identified in the Genetic Investigation of ANthropomorphic Traits (GIANT) consortium [17] of EA studies in association with WHR-BMI (r^2^ = 0.007, D′ = 0.093 among YRI Hapmap participants).

### Further Characterization of *LHX2* and *RREB1* Loci

Given the tendency of waist-associated SNPs to exhibit sex-specific effects in samples of EA [17], we first tested the two AA waist loci for evidence of sexual dimorphism (**Supplementary Table S4**). There was no appreciable difference between the beta coefficients for the lead SNPs at *LHX2* or *RREB1* in women compared to men in the joint analysis of Stage 1 and Stage 2 samples (p_sex difference_ >0.46), suggesting little to no sexual dimorphism with respect to these 2 loci.

**Figure 2:**
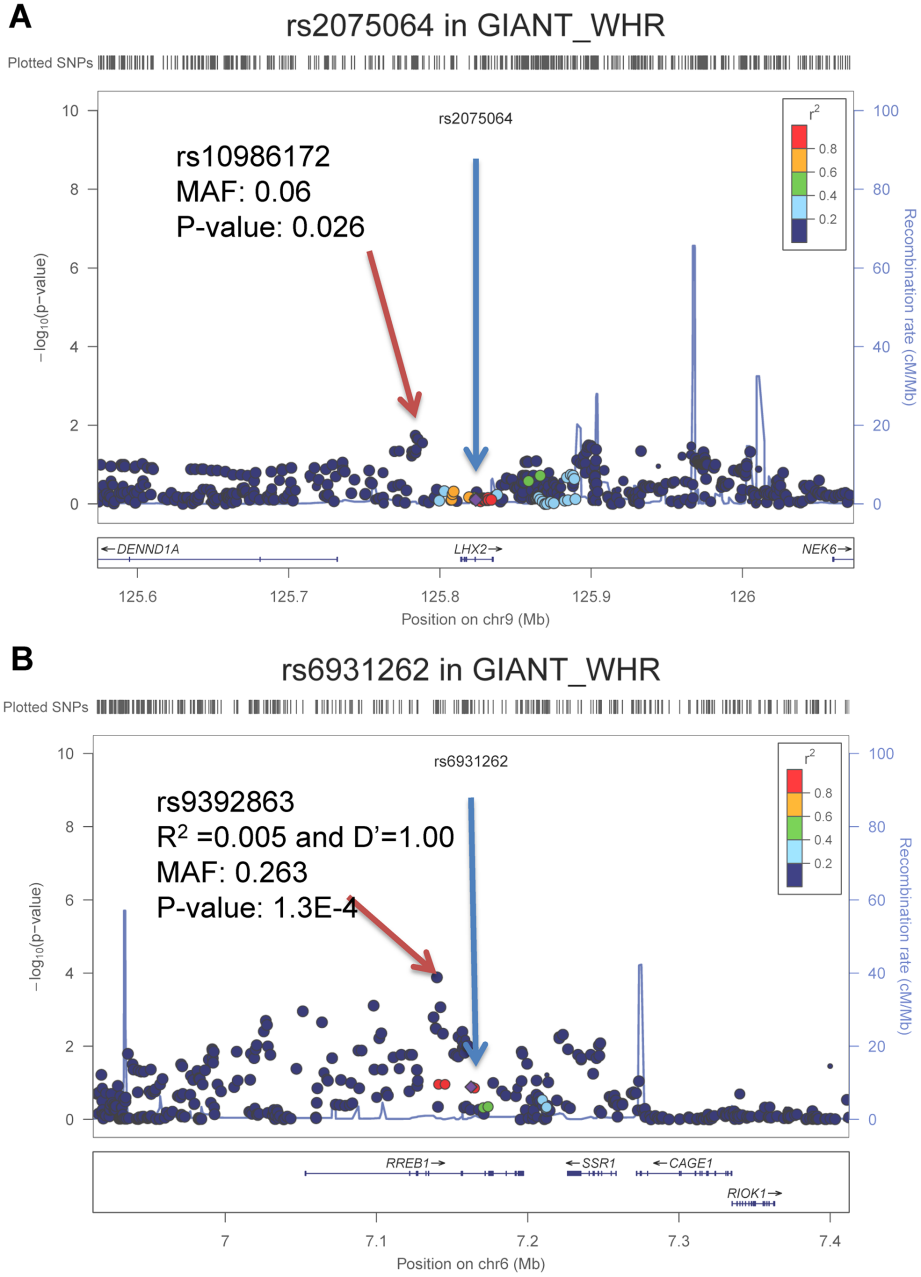
Regional association plots for *LHX2* and *RREB1* in GIANT consortium with participants of European ancestry. The blue arrow points to the index SNPs identified from the samples of African ancestry and red arrow points to the best SNPs in GIANT consortium samples of European ancestry.

Next, we tested whether the loci identified in the samples of African ancestry also demonstrate nominal associations in samples of European ancestry. We interrogated the evidence for association, both directional consistency and statistical significance, of these two SNPs in the GIANT consortium meta-analysis of WHR-BMI (n = 77,167 EA participants, http://www.broadinstitute.org/collaboration/giant/index.php/GIANT_consortium) [17]. Neither rs2075064 at *LHX2* (p = 0.78) nor rs6931262 at *RREB1* (p = 0.13) was statistically significant. The direction of effect for the risk allele was consistent for *RREB1* between EA and AA samples, while it was direction-inconsistent for *LHX2*. However, because linkage disequilibrium patterns with causal SNPs can differ, or allelic heterogeneity can exist across ethnicities, we tested for SNP associations within the 250 kb flanking genomic regions centered at our two top signals to examine whether SNPs in these genomic regions might be associated with WHR-BMI in EA samples. For the *LHX2* region, the SNP with the lowest p-value was rs10986172 (MAF = 0.06, p = 2.6×10^−2^, ∼30 kb from rs2075064; **Figure 2a**), which did not reach the Bonferroni-corrected p-value threshold of 6.02×10^−4^ (0.05/83 independent tests). For the *RREB1* region, the SNP with the lowest p-value in the European Ancestry data was rs9392863 (MAF = 0.26, p = 1.30×10^−4^, ∼20 kb from rs6931262, LD with rs6931262: r^2^ = 0.005 and D′ = 1.00 in HapMap CEU; **Figure 2b**) which met the Bonferroni-corrected threshold of 6.10×10^−4^ (0.05/82) in EA samples. Note that the association for rs9392863 was not significant (p-value = 0.57, LD with rs6931262: r^2^ = 0.001 and D′ = 1.00 in HapMap YRI) in our AA samples.

Waist circumference may be greater in tall adults. To distinguish the evidence of association with WC-BMI from height, we also tested whether rs2075064 at *LHX2* might also be associated with height in the GIANT GWAS of Height (http://www.broadinstitute.org/collaboration/giant/index.php/GIANT_consortium) [23]. No evidence of association was noted (p-value of 0.95).

### Local Ancestry Analysis

Recently admixed individuals, such as samples of African ancestry, may have inherited ancestry from more than one ancestral population. However, local ancestry may be confounded with the association signal and lead to spurious association in association analysis. So to further characterize the differences by ancestral groups of our two novel loci (*LHX2* and *RREB1*), we performed a sensitivity analysis by additionally adjusting for local ancestry to account for the effect on our trait of interest due to the local ancestry at the tested variant using 5 Stage 1 African ancestry studies. Local ancestry adjustment resulted in similar effect estimates (**Supplementary Table S5**), suggesting it is unlikely to account for our reported signals.

### Interrogation of Known European WHR Loci in African Ancestry Participants

**Figure 3:**
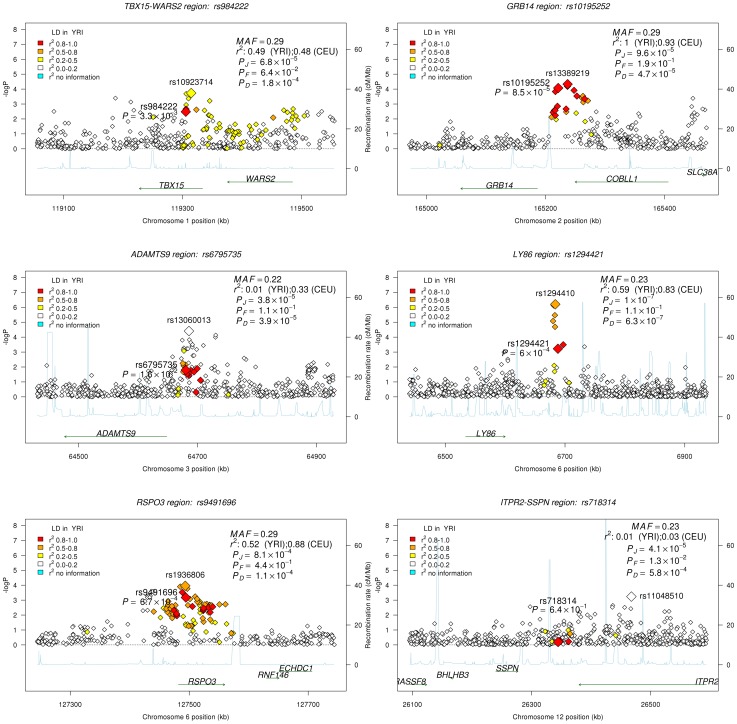
Regional association plots for all confirmed loci from the GIANT locus interrogation. Each figure is centered by the index SNP (big red) with rs-number and p-value information (stage 1 only); another big rectangle is the best SNP in African Americans, with information including MAF = minor allele frequency; linkage disequilibrium information in HapMap YRI and CEU*; P_D_, P_F_, and P_J_* are the single GC-corrected p-value obtained from discovery cohorts only, follow-up cohorts and joint discovery and follow-up data, respectively. Double GC-corrected p-value can be found in **Table 4**.

Given the association of the *RREB1* locus with WHR-BMI in both AA and EA participants, we next examined fourteen previously published loci in association with WHR-BMI in EA participants [17] in our AA sample (**Table 3**). Twelve (except rs6861681 and rs1055144) of the fourteen SNPs had the same effect direction with respect to the beta coefficient (binomial distribution p-value = 0.0065), and five demonstrated nominal significance (p<0.05) in our AA Stage 1 sample (p-value range 1.6×10^−2^ to 8.5×10^−5^). We also conduct two-sample t-test to compare the beta coefficients between EA samples and AA samples. None of these fourteen SNPs displayed significant heterogeneity between the two races. We next interrogated the flanking 250 kb genomic regions centered at each of the 14 SNPs in our AA dataset. Of the 14 SNPs, 9 SNPs met the locus-specific Bonferroni corrected threshold in the Stage 1 sample and were carried forward for Stage 2 validation. In the combined Stage 1 and 2 sample, of these 9 SNPs, 6 remained significant with p-values less than the locus-specific Bonferroni-corrected threshold (0.05 divided by the number of independent SNPs within the flanking region of each index SNP; *TBX15-WARS2*, *GRB14*, *ADAMTS9*, *LY86*, *RSPO3*, *ITPR2-SSPN*, **Table 4**). **Figure 3** presents the regional association plots of these six loci. Except for the *ITPR2-SSPN* region, the five best AA SNPs were in linkage disequilibrium, r^2^>0.3 (LD in HapMap II CEU), with the original index SNPs previously reported in EA participants.

**Table 3:** Examination of index SNPs within known loci in EA in AA for trait WHR ratio adjusted for BMI.

					Index SNP Results													
index SNP information					EA Sample			AA samples					
SNP	chr	bp (b36)	Genes	All^1^	EAF^2^	beta	SE	*P-val*	*N*	beta	SE	EAF^2^	P^4^_Het_
**SNPs associated with waist-related trait at significant level** 3													
rs984222	1	119305366	*TBX15-WARS2*	g/c	0.37	0.04	0.005	3.3E-03	19078	0.03	0.011	0.45	0.66
rs10195252	2	165221337	*GRB14*	t/c	0.60	0.03	0.005	8.5E-05	19654	0.05	0.012	0.28	0.78
rs6795735	3	64680405	*ADAMTS9*	c/t	0.41	0.03	0.005	1.6E-02	19630	0.03	0.014	0.19	0.85
rs1294421	6	6688148	*LY86*	g/t	0.39	0.03	0.005	6.0E-04	19625	0.04	0.012	0.74	0.46
rs9491696	6	127494332	*RSPO3*	g/c	0.52	0.04	0.005	6.7E-04	19642	0.04	0.011	0.39	0.70
rs718314	12	26344550	*ITPR2-SSPN*	g/a	0.74	0.03	0.005	6.4E-01	19637	0.01	0.014	0.19	0.85
**Further SNPs evaluated in follow up but not achieving significance in combined analysis** 3													
rs4846567	1	217817340	*LYPLAL1*	g/t	0.28	0.04	0.005	1.4E-01	19671	0.03	0.019	0.92	0.36
rs6784615	3	52481466	*NISCH-STAB1*	t/c	0.94	0.05	0.010	8.8E-01	14326	0.01	0.040	0.98	0.33
rs6905288	6	43866851	*VEGFA*	a/g	0.56	0.03	0.005	1.0E-01	19609	0.02	0.010	0.50	0.62
**SNP evaluated but not achieving significance in discovery analysis** 3													
rs1011731	1	170613171	*DNM3-PIGC*	g/a	0.57	0.03	0.005	1.4E-01	19480	0.02	0.014	0.82	0.41
rs6861681	5	173295064	*CPEB4*	a/g	0.34	0.03	0.005	4.3E-01	3074	−0.04	0.053	0.07	0.94
rs1055144	7	25837634	*NFE2L3*	t/c	0.21	0.03	0.006	5.4E-01	19606	−0.01	0.022	0.06	0.95
rs1443512	12	52628951	*HOXC13*	a/c	0.24	0.03	0.005	5.5E-02	19644	0.02	0.011	0.57	0.57
rs4823006	22	27781671	*ZNRF3-KREMEN1*	a/g	0.57	0.03	0.005	8.5E-02	19444	0.02	0.011	0.69	0.49

The index SNPs is from Heid et al, Nature Genetics 2010 [17].

1 effect allele/other allele.

2 effect allele frequency.

3 Significance classification refers to the interrogation results of best SNP in **Table 4**.

4 p-value of heterogeneity test of beta between EA and AA samples.

**Table 4:** Interrogation of best SNPs with the smallest p-value within known EA loci in AA for trait WHR ratio adjusted for BMI.

	Best SNP results in AA sample																
	Stage 1										Stage 2			Combined			
Genes	Best_SNP	All^1^	EAF^2^	*P-val*	beta	SE	N^3^	P^4^	YRI^5^ r^2^ (D′)	CEU^5^ r^2^ (D′)	beta	SE	P-val^6^	beta	SE	*P-val*	*P_2GC_* 7
**SNPs associated with waist-related trait at significant level**																	
*TBX15-WARS2*	rs10923714	a/g	0.29	1.8E-04	0.04	0.01	34	1.5E-03	0.49 (1.00)	0.48 (1.00)	0.03	0.02	6.4E-02	0.04	0.01	6.8E-05	1.1E-04
*GRB14*	rs13389219	t/c	0.71	4.7E-05	−0.05	0.01	42	1.2E-03	1.00 (1.00)	0.93 (1.00)	−0.02	0.02	2.0E-01	−0.04	0.01	9.5E-05	1.4E-04
*ADAMTS9*	rs13060013	a/c	0.22	3.9E-05	0.05	0.01	95	5.3E-04	0.01 (0.45)	0.33 (1.00)	0.02	0.02	1.2E-01	0.04	0.01	3.8E-05	5.9E-05
*LY86*	rs1294410	t/c	0.23	6.3E-07	−0.06	0.01	130	3.8E-04	0.39 (0.68)	0.83 (0.96)	−0.02	0.02	1.1E-01	−0.05	0.01	1.0E-06	1.8E-06
*RSPO3*	rs1936806	t/c	0.29	1.1E-04	0.04	0.01	24	2.1E-03	0.52 (1.00)	0.88 (1.00)	0.00	0.02	4.4E-01	0.03	0.01	8.1E-04	1.1E-03
*ITPR2-SSPN*	rs11048510	c/g	0.23	5.8E-04	0.04	0.01	88	5.7E-04	0.00 (0.25)	0.03 (0.19)	0.04	0.02	1.3E-02	0.04	0.01	4.1E-05	6.0E-05
***Further SNPs evaluated in follow up but not achieving significance in combined analysis***																	
*LYPLAL1*	rs2791547	a/t	0.67	2.0E-03	−0.03	0.01	51	9.8E-04	0.00 (0.09)	0.62 (0.95)	−0.01	0.02	3.7E-01	−0.03	0.01	4.9E-03	6.0E-03
*NISCH-STAB1*	rs4687612	a/g	0.95	5.3E-03	−0.07	0.02	17	2.9E-03	N/A	0.01 (1.00)	−0.03	0.04	2.5E-01	−0.06	0.02	6.0E-03	7.4E-03
*VEGFA*	rs1761769	t/g	0.30	3.1E-04	−0.05	0.01	74	6.8E-04	0.12 (0.70)	0.00 (0.06)	0.01	0.02	2.6E-01	−0.03	0.01	5.1E-03	6.3E-03
**SNP evaluated but not achieving significance in discovery analysis**																	
*DNM3-PIGC*	rs4916264	t/c	0.66	4.4E-03	−0.04	0.02	47	1.1E-03	0.06 (1.00)	0.63 (1.00)							
*CPEB4*	rs2659191	a/g	0.88	1.2E-02	0.04	0.02	92	5.4E-04	N/A	0.16 (1.00)							
*NFE2L3*	rs4719818	t/g	0.21	2.1E-03	−0.04	0.01	56	8.9E-04	0.04 (0.42)	0.07 (0.50)							
*HOXC13*	rs7970400	t/c	0.65	4.6E-03	0.03	0.01	62	8.1E-04	0.02 (0.28)	0.02 (0.17)							
*ZNRF3-KREMEN1*	rs16987063	c/g	0.88	1.6E-02	0.06	0.02	47	1.1E-03	0.01 (0.17)	N/A							

The index SNPs are from Heid et al, Nature Genetics 2010 [17]. Note that **Tables 3** and **4** show different information for the same loci (**Table 3** for index SNP and **Table 4** for best SNPs with the smallest p-value).

1 effect allele/other allele.

2 effect allele frequency.

3 number of independent (typed) SNPs interrogated in AA sample.

4 Bonferroni p-value threshold (0.05/N^3^).

5 HapMAP LD information.

6 one-side test p-value.

7 P_2GC_: double GC-corrected p-value.

Because of the close physical proximity of rs6931262 at *RREB1* and rs1294410 at *LY86* (r^2^ 0.01, D′ 0.35 in YRI, 474 kb apart), we performed a conditional analysis in the largest contributing study, the Women's Health Initiative. When the SNPs were tested individually, the beta coefficient for rs6931262 was −0.054 (p = 0.0039), and for rs1294410 was 0.048 (p = 0.0145). In the conditional analysis, the betas and p-values were not numerically different (beta −0.052 for rs6931262 and 0.046 for rs1294410; p-values 0.0056 and 0.0145, respectively).

Given the prior evidence for sexual dimorphism at many of these known loci [17], we tested for evidence of sex differences at the 6 replicating AA SNPs in the joint Stage 1 and Stage 2 samples (**Supplementary Table S4**). We observed little evidence for sexual dimorphism, with the exception of *GRB14*, which demonstrated a stronger effect size in women as compared to men (p-value = 0.02 for the comparison of the beta coefficients).

### Cross-Trait Associations

Given the evidence for association between waist-based traits and other cardiometabolic risk factors in EA individuals [17], we next examined whether there was similar enrichment in AA individuals (**Table 5**). rs13389219 at *GRB14* was associated with HDL-cholesterol (p = 0.014) [24], triglycerides (p = 0.014) [24], and fasting insulin (p = 0.008) [25], while rs13060013 in *ADAMTS9* was associated with HDL-cholesterol (p = 0.0009) [24] and fasting insulin (p = 0.002) [25]. There were nominal associations with related anthropometric traits for rs1936806 at *RSPO3* in association with BMI [26] (p = 0.003), rs2075064 at *LHX2* in association with BMI [26] (p = 0.002), and height (p = 0.02, Christopher Haiman, personal communication).

**Table 5:** Cross-trait associations for novel loci from Stage1 + Stage 2 in participants of African ancestry.

				BMI (n = 39137)		HDL-C (n = 7813)		LDL-C (n = 7565)		TG (n = 7717)		Glucose (n = 5984)		Insulin (n = 5969)		Height (n = 20427)	
SNP	Gene	All^1^	Beta^2^	Beta	*P-val*	Beta	*P-val*	Beta	*P-val*	Beta	*P-val*	Beta	*P-val*	Beta	*P-val*	Beta	*P-val*
**SNPs from genome-wide association Analysis**																	
rs2075064	LHX2	t/c	−0.070	−0.037	2.1E-03	0.005	8.5E-01	0.023	3.9E-01	−0.005	8.5E-01	0.001	9.4E-01	−0.029	8.9E-02	−0.039	2.3E-02
rs6931262	RREB1	t/c	0.059	−0.013	1.6E-01	0.002	9.3E-01	−0.032	1.1E-01	0.026	1.8E-01	0.008	5.0E-01	−0.015	2.6E-01	−0.012	2.7E-01
**SNPs from cross-population interrogation**																	
rs10923714	*TBX15-WARS2*	a/g	0.038	−0.011	2.1E-01	−0.018	3.1E-01	0.028	1.2E-01	0.015	4.0E-01	−0.002	8.1E-01	−0.008	5.3E-01	0.011	2.9E-01
rs13389219	*GRB14*	t/c	−0.039	0.015	9.0E-02	0.046	1.4E-02	−0.002	9.4E-01	−0.046	1.4E-02	−0.014	1.8E-01	−0.033	7.9E-03	−0.003	7.9E-01
rs13060013	*ADAMTS9*	a/c	0.044	−0.005	6.0E-01	−0.066	8.6E-04	0.001	9.5E-01	0.022	2.8E-01	0.013	2.3E-01	0.040	2.2E-03	0.001	9.2E-01
rs1294410	*LY86*	t/c	−0.051	0.009	3.5E-01	0.010	6.2E-01	0.001	9.6E-01	0.027	1.7E-01	0.006	6.0E-01	0.005	6.8E-01	0.012	2.8E-01
rs1936806	*RSPO3*	t/c	0.033	−0.026	2.8E-03	−0.016	3.8E-01	0.018	3.4E-01	0.026	1.5E-01	0.013	2.1E-01	0.011	3.7E-01	−0.012	2.6E-01
rs11048510	*ITPR2-SSPN*	c/g	0.045	0.000	9.7E-01	0.023	2.7E-01	0.018	3.9E-01	0.008	7.2E-01	0.000	1.0E+00	0.014	3.3E-01	−0.002	8.5E-01

1 effect allele/other allele.

2 effect size based on Stage 1 and Stage 2 combined sample.

## Discussion

We identified 2 loci at *LHX2* and *RREB1* with p<5.0×10^−8^ under single GC-correction for waist-based traits in African ancestry individuals, which were not genome-wide significant (p = 6.5×10^−8^ and p = 5.7×10^−8^) with double-GC correction. Population sub-structure may cause spurious associations in genome-wide association studies and GC factors calculated from variants across the genome are conventionally used to scale the test statistic [27]. However, this method was originally proposed under the hypothesis that only a small number of causal variants underlie complex traits. Recent studies have shown that as the number of causal variants increases, more SNPs (in LD with causal variants) will depart from the null distribution even in the absence of population sub-structure [22], [23]. Furthermore, the GC factor is a function of sample size under a constant phenotypic heritability. Therefore, double GC-correction in a large meta-analysis is likely overly conservative. Thus, we report both single and double GC-corrected values. rs6931262 at *RREB1* is in a region previously identified by the GIANT consortium in European ancestry individuals, although in low linkage disequilibrium with the variant identified in the present study. Interrogation of 14 regions previously identified by the GIANT consortium identified 6 additional SNPs associated with waist traits in AA participants. Two of these loci at *GRB14* and *ADAMTS9* were also associated with metabolic traits in AA. Finally, we observed nominal evidence for sexual dimorphism.

These findings support prior GWAS findings that there are genetic loci for body fat distribution which are distinct from loci associated with generalized adiposity. The GIANT consortium identified 14 loci in association with WHR adjusted for BMI, and the majority was not associated with BMI [17]. Similarly, a SNP at *IRS1* was identified in association with body percent fat that was not associated with generalized adiposity [18]. More recent GWAS of ectopic fat depots have also identified unique loci in association with fatty liver [19], visceral abdominal fat [20], and pericardial fat [21]. The results from the present analysis extend these observations to AA populations, a group at increased risk for obesity and its complications.

Our findings add to the growing appreciation that SNPs correlated with body fat distribution are also associated with metabolic traits [28]. This finding contrasts with genetic loci for BMI, which generally have not been shown to be associated with metabolic traits [29]. In contrast to prior work [17], [18], [20], we observed little evidence for sexual dimorphism in the present analysis, with the exception of *GRB14*. Whereas the GIANT consortium observed stronger effect sizes in women than men among the majority of the 14 WHR SNPs they identified, we observed more modest gender differences and in some instances, the effect size was actually stronger in men as compared to women (*ITPR2-SSPN* and *LHX2*). This raises several hypotheses that warrant some speculation as to why we did not observe similar sexual dimorphism in AA sample as observed in our prior work in EA samples. First, biologically, associations linking the SNPs and gene regions to body fat distribution traits may be different between women and men of AA as compared to EA. Second, in terms of methodology, the traits themselves may be measuring different phenotypic elements of body fat distribution or muscle mass in women as compared to men. Abdominal adipose composition may vary more in EA than AA as EA have greater visceral adipose tissue than AA of similar gender. Finally, statistically, for some loci (*RSPO3* and *ADAMTS9*), we cannot rule out the presence of modest gender differences given the relatively small sample sizes as compared with other analyses. This is further reinforced by our power analysis to detect sex-specific associations. We conducted this power analysis for a common variant (specifically, MAF of 0.25 here) with an effect size difference of 0.054, which is derived from the largest effect difference indicated in Table 2 of Heid et al [17]. Using these assumptions, we have only 6.7% power to detect the variant (MAF of 0.25) with a sample size of 23564 in our discovery stage and 10.9% power with combined sample size 33738 from stage 1 and stage 2. This suggests that we have limited power to detect sexual dimorphism if it indeed exists.

It is notable that our top SNP at *RREB1* is within 1 Mb of *LY86* (lymphocyte antigen 86), one of the 14 novel loci identified by the GIANT genome-wide association meta-analysis of WHR [17]. Given the low pairwise linkage disequilibrium and lack of change in the association of the beta coefficient upon conditional analysis in this region, it is likely that there are two independent genetic effects in this chromosomal region. It is also possible that these two SNPs may be partially tagging an untyped variant that explains the underlying association [30]–[32]. Therefore, fine mapping or deep sequencing of this region is needed and may prove relevant to both AA and EA. Our findings also demonstrate the similarity in the genetic architecture of waist related traits in EA and AA, as 12 of the 14 previously identified WHR loci demonstrated direction-consistent effect estimates in AA as compared to EA participants. For five of these signals (*TBX15-WARS2*, *GRB14*, *ADAMTS9*, *LY86*, *RSPO3*), upon interrogation of the 250 kb flanking region of the index signal, we identified a better proxy (r^2^>0.3 in CEU dataset) SNP of the presumably underlying biologically important alleles at these loci. Indeed, these findings may help improve localization of the true association signal.

rs2075064 is in linkage disequilbrium with variants in the *LHX2* and *DENND1A* genes. *LHX2* is a member of the LHX protein family, the largest group of LIM-domain proteins. LHX proteins are primarily transcriptional regulators, with a known role in tissue-specific gene expression. They take part in the determination of cell lineage and identity in a wide range of tissues, including the adipocyte differentiation of human adipose-derived stem cells [33]. Variants in *DENND1A* gene have been associated with polycystic ovarian syndrome in both European ancestry [34] and Chinese women [35]. Taken together, these findings highlight how future studies can further our understanding of how genes in this region may contribute to body fat distribution and related obesity phenotypes.

There are several genes in the region of rs6931262. *RREB1* (Ras-responsive element binding protein 1) participates in Ras signaling and cancer progression in bladder cancer [36] prostate cancer [37], and melanoma [38]. *RREB1* is not known to play a role in adipose tissue, and SNPs in this gene have previously been associated with serum urate levels [39]. *SSR1*, *CAGE1*, and *RIOK1* are also located in this genomic region, although SNPs mapping to these genes do not appear to be in linkage disequilibrium with the SNP cluster of interest.

There are several potential implications of this work. First, these analyses highlight how novel loci for body fat distribution, above and beyond generalized adiposity, can be elucidated by performing GWAS in diverse ethnic populations. Second, we demonstrate some important similarities in the associations among AA as compared to EA individuals with regards to the loci uncovered as well as pleiotropy with other cardio-metabolic phenotypes. Finally, while many of the beta coefficients were similar in women as compared to men, we did uncover modest evidence for sexual dimorphism in the present AA sample.

A major strength of this study is the large sample size of AA participants, representing the largest study to date for waist-based phenotypes in AA. This study has similar limitations to other GWAS performed in AA populations. While the overall sample size was large, the discovery sample was still considerably smaller than those for GWAS meta-analysis conducted in samples of primarily EA populations. In the present analysis, to have 80% power to detect an association that explains 0.1% of the trait variance at a MAF of 0.25 would require 39581 participants. With our largest WC sample size (n = 23564) in discovery stage, we have only 28% power to detect common variant explaining 0.1% of the variance of WC. In addition, GWAS panels such as the Affymetrix 6.0 chip were largely designed based on EA populations and have more limited SNP coverage for AA samples. For example, one analysis of 76 genes reported that only approximately 45–55% of SNPs were tagged (*r* ^2^>0.8) on the Affymetrix 6.0 panel in YRI samples [40]. Kang and colleagues [41] demonstrated that both local and global ancestry estimates similarly attenuated spurious results due to population stratification in their study of AA ancestry individuals. As with all studies in admixed populations, while association analyses were adjusted for global population structure using principal components, there may be residual population substructure leading to false positive results. Given the minimal attenuation that we observed with local ancestry adjustment, our key findings are unlikely to be spurious. We performed 12 analyses, raising the possibility of false positive findings using standard significance thresholds. *PCSK1* is a bona fide locus for obesity [42], yet this SNP failed to replicate in our findings. While we can not rule out power as the reason for the lack of replication, this signal may also have represented a false positive finding in our dataset. Heterogeneity between study samples may limit power, but this is an issue in GWAS and not unique to the present investigation. After double GC correction, our findings did not reach genome-wide significance. However, double GC correction may be overly conservative [22], [23]. Finally, a general limitation in GWAS is that coverage of rare (MAF<1%) and low frequency (1%<MAF<5%) variants is poor, and thus associations with rarer variants are likely missed.

GWAS of body fat distribution traits in a large AA sample has revealed two loci likely associated with fat distribution, as well as nominal evidence for association at 6 loci previously identified among EA individuals. These findings highlight similarities and differences in the genetic architecture of body fat distribution in AA and EA individuals, and reinforce the concept that there are loci for fat distribution above and beyond generalized adiposity.

## Materials & Methods

### Phenotype Definition

We analyzed waist-based traits including waist circumference (WC) and waist-hip ratio (WHR), a measure of body fat distribution [43]. Details regarding trait acquisition within each cohort can be found in the Text S1. Individuals less than 20 years of age were excluded from all analyses. Within each cohort, we created two sets of residuals for WC and WHR, one adjusted for age, age^2^, study site (if applicable) and another additionally adjusted for BMI. Analyses were conducted separately for men and women. The raw residuals were then transformed through an inverse normal function for each subgroup and these transformed residuals were used as our phenotypes in the association analyses. The cohorts with related individuals additionally performed sex-combined analysis. We analyzed four phenotypes: waist circumference (WC), waist circumference adjusted for BMI (WC-BMI), waist hip ratio (WHR) and waist hip ratio adjusted for BMI (WHR-BMI).

### Samples

We conducted analysis of WC and WHR in up to 33738 and 27489 AA individuals, respectively. Specifically, the analysis included for WC up to 23,564 individuals and WHR up to 19,744 individuals in stage 1 while 10,174 AA individuals with WC and 7,745 AA individuals with WHR in stage 2. Stage 1 cohorts were part of the CARe consortium and other cohorts that were identified with GWAS data at the time the study started. Stage 2 cohorts with in silico GWAS data were identified later. Some participating studies, including CFS, Family Heart Study, GENOA, HUFS, HyperGEN, GeneSTAR, JHS, MESA-family and SIGNET (REGARDS, SUGAR), are family studies. The CARe consortium (ARIC, CARDIA, CFS, JHS, MESA) consists of several population-based studies that included African ancestry individuals. The WHI study was a clinical trial. HANDLS is a community-based study. Family Heart Study is a multicenter family-based study. GeneSTAR is a prospective study of vascular diseases. GENOA and HYPERGEN are cohorts of sibships enriched for hypertension. Health ABC is a random sample of Medicare beneficiaries in and surrounding Pittsburgh, Pennsylvania, and Memphis, Tennessee. HUFS is a population-based family study in the Washington, D.C. metropolitan area. MEC is a prospective cohort study including a nested breast cancer case-control study. WCHS is a case-control study of breast cancer in the New York City and New Jersey. CBCS is population-based case-control study on Breast cancer. Both MEC and MDA are Prostate cancer case-control studies. Black Women's Health Study is an ongoing follow-up study of 59,000 African American women from across the U.S. CHS is a population-based study of risk factors for CHD and stroke. REGARDS is an observational cohort and SUGAR is a community based family studies focusing on Type 2 Diabetes. Each participating study has obtained institutional review board approval on research involving human subjects and all subjects provided written informed consent. Details regarding each cohort can be found in the Text S1.

### Genotyping and Imputation

Genotype information for each cohort is presented in **Supplementary Table S2**. As shown in **Table 1**, the genetic ancestry of our samples, African American, is also partly from European Ancestry. Simply using all YRI sample as reference panel would be inappropriate, given that we generally see an average of 20% CEU admixture. To better capture the genetic structure of our samples, all the genotypes from discovery cohorts were imputed using combined HapMap 1∶1 CEU+YRI as reference panel. This imputation has resulted in an allelic concordance rate of 95.6%, which is compatible to rates calculated with the HapMap 2 YRI individuals [44]. Our follow-up stage (Stage 2) included in silico and de novo follow-up cohorts. In-silico follow-up studies similarly use the combined CEU+YRI in HapMap as their reference panel for genotype imputation. Then the expected allele dosage was used in the association analysis to account for the uncertainty introduced by the genotype imputation. More details regarding imputation were in **Supplementary Table S2**.

### Statistical Methods for Discovery (Stage 1)

In each discovery study, genome-wide tests for association between SNPs and phenotypes were conducted separately for men and women using linear regression with principal components adjustment to adjust for global population substructure. In studies from families, men and women were also combined for analyses. Linear mixed effect models, where appropriate, were used to account for the relatedness in family studies.

In addition to study-specific filters, a centralized quality control procedure was performed to extensively examine and check all study-specific results files before meta-analysis. We examined the plausible values for all reported summary statistics to check for potential errors. The genomic control lambda (λ) value for each set of results was checked for potential p-value inflation. We analyzed SNPs with imputation quality scores greater than 0.3 for studies using MACH or BimBam software, and greater than 0.4 for studies that used other imputation software such as IMPUTE. Additionally, we filtered out SNPs where the minor allele frequency times the number of subjects was smaller than or equal to 5, to ensure robust estimates.

### Meta-Analysis

We performed fixed-effects meta-analyses of study-specific genome-wide association results using the inverse-variance weighted approach for the traits described above. Three sets of meta-analyses were conducted for each phenotype using (1) men only results, (2) women only results, and (3) joint men-only and women-only results for studies of unrelated individuals, and sex-combined results for studies with related individuals. The calculated λ genomic control (GC) correction was applied to each cohort's result. Recent studies showed that under polygenic inheritance, test statistics in large meta-analyses are expected to be elevated even when there is no population sub-structure [22], [23]. To avoid an overly conservative adjustment, we focused on the single GC-corrected result. However, we also report the double GC-corrected p-value for the joint meta-analysis of the stage 1 and stage 2 samples.

### Local Ancestry Analysis

As a sensitivity analysis, we assessed the impact of local ancestry by including SNP specific local ancestry estimates as a covariate in models for genome-wide significant signals in both the CARE and WHI studies. Locus-specific ancestry (i.e. probabilities of whether an individual has 0, 1, or 2 alleles of African ancestry at each locus) was only available for directly genotyped SNPs and was estimated using a Hidden Markov Model and the local haplotype structure to detect transitions in ancestry along the genome [45]. We considered signals robust to adjustment for local ancestry when the Beta was numerically similar.

### Interrogation of GIANT Loci in the Samples of African Ancestry

We applied a procedure to evaluate the transferability of association signals across different ethnicities [46]. Specifically, in addition to validating the previously reported index SNPs identified in studies of EA participants [17], we interrogated the surrounding genomic regions in our AA samples. For each reported index EA SNP, we first examined the results in our AA samples and tested for consistency of direction, with respect to the beta coefficients of index SNPs, between EA and AA samples. To accommodate the difference of LD structure across ethnicities, we then interrogated ±250 kb regions around the index SNPs and identified the SNP with the smallest p-value in AA within the interrogated genomic region. The loci-specific significance threshold was based on Bonferroni correction, defined as 0.05 divided by the number of independent SNPs within an interrogated region. SNPs meeting genome-wide (p<5.0×10^−8^) or suggestive (5.0×10^−8^<p<5.0×10^−6^) in the Stage 1 meta-analysis were carried forward for follow-up in Stage 2 and joint Stage 1 and 2 meta-analysis. To test the consistency of effect directions between AA and EA samples, a p-value was calculated based on the cumulative binomial distribution for the observed or more extreme number of variants with a consistent direction.

### Follow-up Analysis (Stage 2) and Joint Analysis of Discovery and Follow-up (Stage 1 and 2) Data

An analysis approach consistent with the discovery stage (ie Stage 1), described above, was used for the Stage 2 studies. In this stage, the variants of interest identified from our analysis of Stage 1 and interrogation of previously published EA WHR-BMI loci were followed up in different samples for follow-up meta-analysis and confirming the association. We then conducted additional joint meta-analysis, including studies from both Stage 1 and Stage 2 discovery and follow-up data. In Stage 2 analysis, the replication was defined as having a beta coefficient consistent with the discovery stage; follow-up p-values are thus represented as one-sided tests. For the joint analysis, we used the standard threshold of p-value<5×10^−8^ for genome-wide significance and a locus-specific Bonferroni corrected threshold for the regions identified by the GIANT consortium.

For newly identified SNPs from both genome-wide association analyses and previous region interrogation analyses, we performed sex-specific association analyses and also tested the difference of meta-analyzed sex-specific beta-estimates (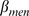 and 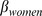) using the t-statistic
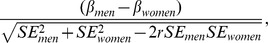
where *r* is the Spearman rank correlation coefficient between 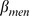 and 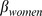 across all SNPs. Note that we are comparing two parameters and testing whether their difference is equal to zero. This is basically the setting of a two-sample test. Although based on our sample size (n>5000 in combined analysis), the Z-statistic should work well due to the Central Limit Theory. However, we intended to conservatively use the t-statistic here to calculate the p-value.

### Interrogation of Novel AA Loci in GIANT

We also examined the GIANT consortium results [17] for evidence of association for the novel loci identified in the AA samples. We applied similar interrogation procedures detailed in the previous section of the Interrogation of GIANT Loci in the samples of African Ancestry. Briefly, we first looked up the association results for the AA index SNP in GIANT and followed up with the interrogation of its ±250 kb flanking region. The significance was evaluated as 0.05 divided by the number of independent variants within the interrogated region.

### Cross-Trait Analyses

For the newly identified SNPs from both GWAS and the interrogation analysis we performed cross-trait association analyses of metabolic risk factor and related anthropometric measures, including BMI [26] , HDL-cholesterol [24], LDL-cholesterol [24], triglycerides [24], glucose and insulin [25], and height (Christopher Haiman, personal communication), in AA samples for the newly identified SNPs from both genome-wide association analysis and the interrogation analysis.

## Supporting Information

Figure S1 Figure S1a – Quantile-quantile and Manhattan plots for genome-wide association for waist circumference and waist circumference adjusted for BMI in sex-specific samples and all samples. Figure S1b – Quantile-quantile and Manhattan plots for genome-wide association for waist-hip ratio and waist-hip ratio adjusted for BMI in sex-specific samples and all samples.
(DOC)

Table S1 Study sample characteristics by gender.
(DOC)

Table S2 Genotyping and imputation platforms used by all participating studies.
(DOC)

Table S3 Imputation quality at 8 associated loci.
(DOC)

Table S4 Gender-specific association analysis at 8 associated loci.
(DOC)

Table S5 Local ancestry analysis for two top variants (rs2075064 at *LHX2* and rs6931262 at *RREB1*) with single GC-corrected p-value<5×10^−8^.
(DOC)

Text S1 Study specific methods.
(DOC)
