# Supplementary material for: Genome-Wide Association of Body Fat Distribution in African Ancestry Populations Suggests New Loci
Source: PLoS Genet. 2013 Aug 15;9(8):e1003681. doi: 10.1371/journal.pgen.1003681 (PMC3744443; doi:10.1371/journal.pgen.1003681)

**Supplementary Figures**

**Figure S1a** – Quantile-quantile and Manhattan plots for genome-wide association for waist circumference and waist circumference adjusted for BMI in sex-specific samples and all samples.


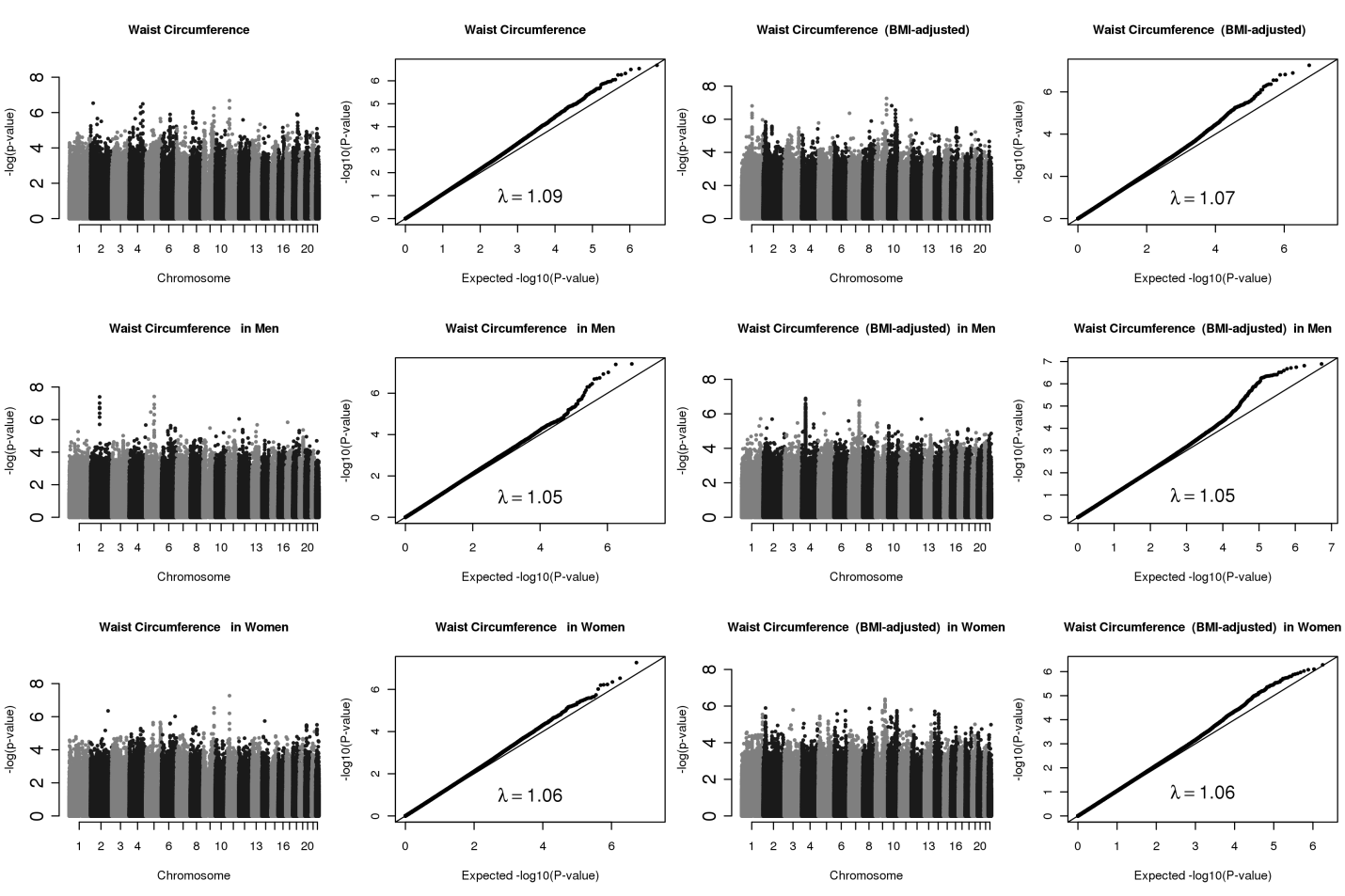


**Figure S1b** – Quantile-quantile and Manhattan plots for genome-wide association for waist-hip ratio and waist-hip ratio adjusted for BMI in sex-specific samples and all samples.


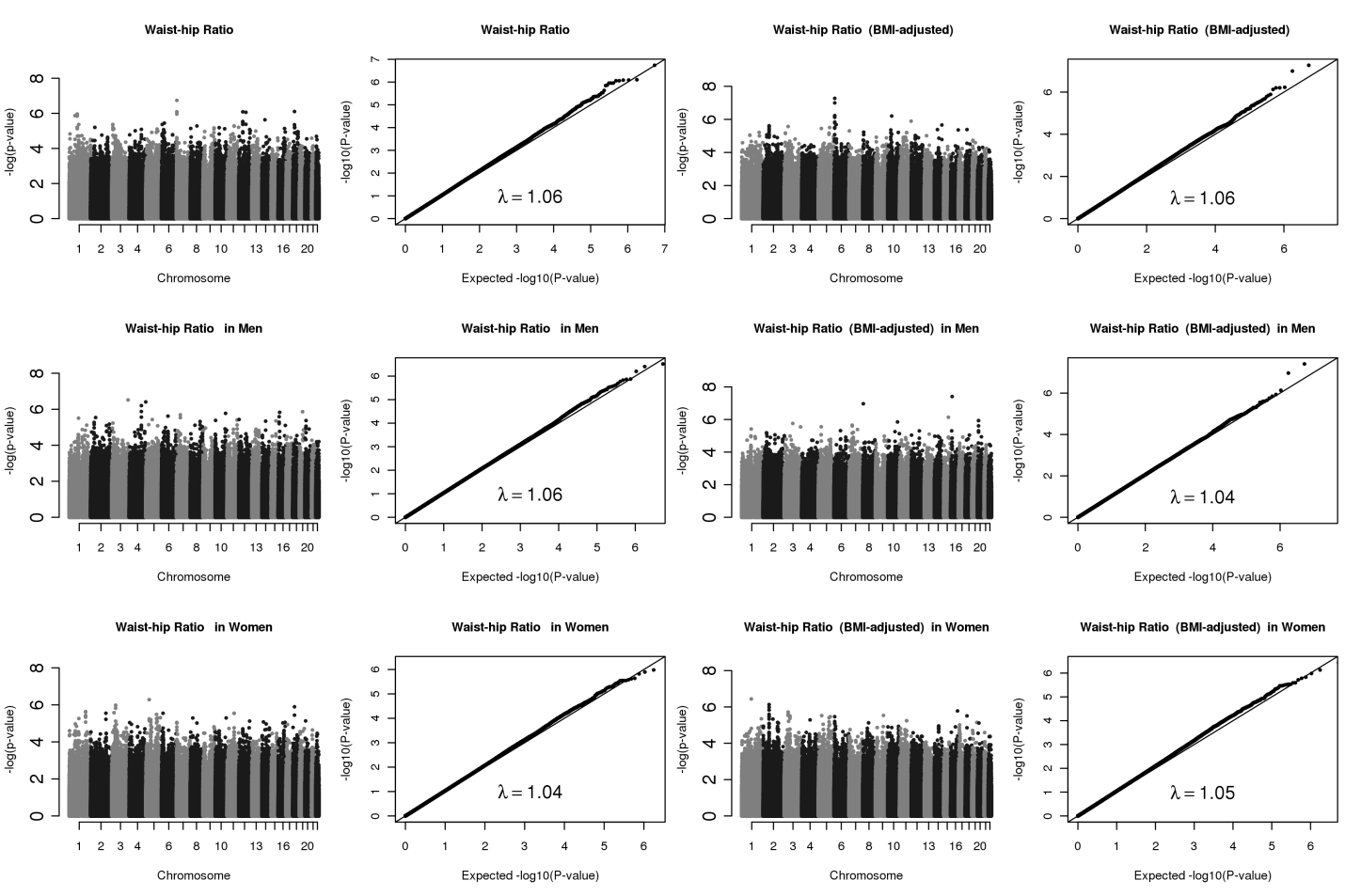

Supplement: Figure S1 — a – Quantile-quantile and Manhattan plots for genome-wide association for waist circumference and waist circumference adjusted for BMI in sex-specific samples and all samples. Figure S1b – Quantile-quantile and Manhattan plots for genome-wide association for waist-hip ratio and waist-hip ratio adjusted for BMI in sex-specific samples and all samples. (DOC) [file pgen.1003681.s001.doc]
