# Supplementary material for: Genome-Wide Association of Body Fat Distribution in African Ancestry Populations Suggests New Loci
Source: PLoS Genet. 2013 Aug 15;9(8):e1003681. doi: 10.1371/journal.pgen.1003681 (PMC3744443; doi:10.1371/journal.pgen.1003681)
Supplement: Table S1 — Study sample characteristics by gender. (DOC) [file pgen.1003681.s002.doc]

**Supplementary Table S1. Study Sample Characteristics by Gender**
