# Supplementary material for: Genome-Wide Association of Body Fat Distribution in African Ancestry Populations Suggests New Loci
Source: PLoS Genet. 2013 Aug 15;9(8):e1003681. doi: 10.1371/journal.pgen.1003681 (PMC3744443; doi:10.1371/journal.pgen.1003681)
Supplement: Table S2 — Genotyping and imputation platforms used by all participating studies. (DOC) [file pgen.1003681.s003.doc]

**Supplementary Table S2**- Genotyping and Imputation Platforms Used by all Participating Studies

|  | **Array type** | **Genotype calling** | **QC filters for genotyped SNPs used for imputation** | **No of SNPs used for imputation** | **Imputation** | **Imputation Backbone for phased CEU haplotypes (NCBI build)** | **Filtering of imputed genotypes** | **Data management and statistical analysis** |
| --- | --- | --- | --- | --- | --- | --- | --- | --- |
| **CARE Consortium** | Affymetrix 6.0 | Birdseed v1.33 | all chip QC + pi_hat 0.05 for rate step | 763537 to 846628 | MACH, 2 rounds | combined CEU+YRI reference panel | MAF 1%, rsq_hat 0.3 | plink –-dosage |
| HANDLS | Illumina 1M SNP coverage (709 samples using Illumina 1M and 1Mduo arrays, the remainder using a combination of 550K, 370K, 510S and 240S to equate the million SNP level of coverage) | Illumina GenomeStudio | All chip QC as per readme | 907763 | MACH, 2 rounds | combined CEU+YRI reference panel | None | R, MACH2qtl V1.08 |
| MESA/SHARe | Affymetrix 6.0 | Birdseed v1.33 |  | 861124 | IMPUTE V2 | HapMap Phase I and II - CEU+YRI+CHB+JPT as the reference panel (release #22 - NCBI Build 36 (dbSNP b126)) | None | R |
| Health ABC | Illumina 1M | BeadStudio v3.3.7 | minor allele frequency ≥ 1%, call rate ≥97% and HWE p≥10^-6^ | 1,007,948 | MACH v1.0.16 | 1:1 mixture of CEPH:Yoruban (YRI) reference panel (release 21, build 36) | None | R |
| GENOA | Affymetrix 6.0 & Illumina 1M | Birdseed & GenomeStudio | All SNPs passed genotype QC | 780156 | MACH, 1 round | combined CEU+YRI reference panel HapMap release #22 build 36 | None | R |
| GeneSTAR | Illumina Human 1Mv1_C | Illumina GenomeStudio | HWE p <1e-6, MAF<1%, not present on HapMap | 687132 | MACH v1.0.16, 2 rounds | combined CEU+YRI reference panel | None | R, lmekin function in kinship package |
| Family Heart Study | Illumina Human 1M-DuoV3 array | BEADSTUDIO-GENCALL v3.0 | MAF<1%, HWE p<1E-06, call rate >98%, removed SNPs not in HapMap | 754,504 | MACH v1.0.16 | CEU+YRI from HapMap (release 21, build 36) | None | SAS, R, MACH |
| HyperGEN | Affymetrix Genome-Wide Human SNP Array 6.0 | Birdseed v1.33 | All chip QC as per readme | 846813 | MACH v1.0.16 | Build 36, Phase I+II data, release 22, using the ‘revised union’ of CEU+YRI SNPs | MAF 1%, rsq_hat 0.3 | plink –-dosage |
| HUFS | Affymetrix 6.0 | Birdseed v2 | MAF 1%, SNP call rate 95%, sample call rate 95% | 842152 | MACH, 2 rounds | CEU and YRI reference panels | MAF 1%, SNP call rate 90%, HWE 1e-3, genotypic concordance 95% | PLINK, SAS |
| WHI | Affymetrix 6.0 | Birdseed v1.33 | MAF 1%, SNP call rate 95%, sample call rate 95% | 829,370 | MACH v1.0.16 | HapMap phase 2 release 22 build 36 and the HapMap Reference panel 1:1 CEU:YRI phase II. |  | PLINK, ProbABEL |
| CHS | Illumina HumanOmni1-Quad_v1 BeadChip system | Illumina GenomeStudio | call rate <97%, heterozygotes=0, pHWE<10E-5, >1 duplicate error or Mendelian Inconsistency | 963248 | BEAGLE 3.2.1 | The data were imputed to HapMap Phase III using reference panels from the ASW, YRI and CEU panels and separately imputed using HapMap Phase II using the CEU and YRI reference panels (build 36). | dosage variance<0.01 | R |
| SIGNET | Affymetrix 6.0 | Birdseed v2 | chipQC,callrate,Mend Err | 843739 | MACH, 2 rounds | CEU and YRI reference panels | MAF 1%,Rsq 0.4 | MERLIN |
