# Supplementary material for: Genome-Wide Association of Body Fat Distribution in African Ancestry Populations Suggests New Loci
Source: PLoS Genet. 2013 Aug 15;9(8):e1003681. doi: 10.1371/journal.pgen.1003681 (PMC3744443; doi:10.1371/journal.pgen.1003681)
Supplement: Table S3 — Imputation quality at 8 associated loci. (DOC) [file pgen.1003681.s004.doc]

**Supplementary Table S3 Imputation quality^1^ at 8 associated loci**

|  | rs2075064 | rs6931262 | rs10923714 | rs13389219 | rs13060013 | rs1294410 | rs1936806 | rs11048510 |
| --- | --- | --- | --- | --- | --- | --- | --- | --- |
| WHI/SHARe | 0.90 | 0.96 | 1.00 | 0.98 | 0.99 | 1.00 | 1.00 | 0.87 |
| HANDLS | 1.00 | 1.00 | 1.00 | 1.00 | 0.99 | 0.94 | 0.90 | 0.98 |
| MESA/SHARe (Family) | 1.00 | 0.97 | 1.00 | 1.00 | 0.98 | 1.00 | 1.00 | 0.91 |
| Health ABC | 1.00 | 1.00 | 1.00 | 1.00 | 0.99 | 0.95 | 0.97 | 0.98 |
| GENOA | 0.77 | 0.91 | 1.00 | 0.97 | 0.98 | 0.94 | 0.92 | 0.86 |
| GeneSTAR | 1.00 | 1.00 | 1.00 | 0.99 | 0.96 | 0.93 | 0.95 | 0.96 |
| Family Heart Study | 1.00 | 1.00 | 1.00 | 1.00 | 0.99 | 0.94 | 0.97 | 0.99 |
| HyperGEN | 0.77 | 0.95 | 1.00 | 0.96 | 0.99 | 1.00 | 1.00 | 0.82 |
| HUFS | 0.85 | 0.95 | 1.00 | na | 0.98 | 1.00 | 1.00 | 0.92 |
| CARe Studies* |  |  |  |  |  |  |  |  |
| ARIC | 0.90 | 0.93 | 1.00 | 1.00 | 0.96 | 1.00 | 1.00 | 0.92 |
| CARDIA | 0.82 | 1.00 | 1.00 | 0.99 | 1.00 | 1.00 | 1.00 | 0.83 |
| CFS | 0.88 | 0.97 | 1.00 | 0.98 | 0.99 | 1.00 | 0.99 | 0.86 |
| JHS | 0.87 | 0.95 | 1.00 | 0.98 | 0.96 | 1.00 | 1.00 | 0.84 |
| MESA | 0.93 | 1.00 | 1.00 | 0.95 | 0.99 | 1.00 | 1.00 | 0.81 |
| **Replication cohort** |  |  |  |  |  |  |  |  |
| CHS | 1.00 | 1.00 | 1.00 | 1.00 | 1.00 | 1.00 | 1.00 | 1.00 |
| BWHS | na | na | 1.00 | 1.00 | 1.00 | 1.00 | 1.00 | 1.00 |
| REGARDS_CASE | 0.94 | 0.97 | 1.00 | 0.98 | 0.99 | 1.00 | 1.00 | 0.90 |
| REGARDS_CONTROL | 0.94 | 0.97 | 1.00 | 0.98 | 0.99 | 1.00 | 1.00 | 0.90 |
| SUGAR_CASE | 0.94 | 0.97 | 1.00 | 0.98 | 0.99 | 1.00 | 1.00 | 0.90 |
| SUGAR_CONTROL | 0.94 | 0.97 | 1.00 | 0.98 | 0.99 | 1.00 | 1.00 | 0.90 |
| CBCS Breast Cancer Cases | 1.00 | 1.00 | 1.00 | 1.00 | 0.99 | 0.95 | 0.96 | 0.98 |
| CBCS Breast Cancer Controls | 1.00 | 1.00 | 1.00 | 1.00 | 0.99 | 0.95 | 0.96 | 0.98 |
| MEC Breast Cancer Cases | 1.00 | 1.00 | 1.00 | 1.00 | 0.99 | 0.95 | 0.96 | 0.98 |
| MEC Breast Cancer Controls | 1.00 | 1.00 | 1.00 | 1.00 | 0.99 | 0.95 | 0.96 | 0.98 |
| MEC Prostate Cancer Cases | 1.00 | 1.00 | 1.00 | 1.00 | 0.99 | 0.95 | 0.96 | 0.98 |
| MEC Prostate Cancer Controls | 1.00 | 1.00 | 1.00 | 1.00 | 0.99 | 0.95 | 0.96 | 0.98 |
| MDA Prostate Cancer Cases | 1.00 | 1.00 | 1.00 | 1.00 | 0.99 | 0.95 | 0.96 | 0.98 |
| MDA Prostate Cancer Controls | 1.00 | 1.00 | 1.00 | 1.00 | 0.99 | 0.95 | 0.96 | 0.98 |
| WCHS Breast Cancer Cases | 1.00 | 1.00 | 1.00 | 1.00 | 0.99 | 0.95 | 0.96 | 0.98 |
| WCHS Breast Cancer Controls | 1.00 | 1.00 | 1.00 | 1.00 | 0.99 | 0.95 | 0.96 | 0.98 |
| ^1^ Imputation value of 1.00 indicates the typed snp used for analysis | | | |  |  |  |  |  |
