# Supplementary material for: Genome-Wide Association of Body Fat Distribution in African Ancestry Populations Suggests New Loci
Source: PLoS Genet. 2013 Aug 15;9(8):e1003681. doi: 10.1371/journal.pgen.1003681 (PMC3744443; doi:10.1371/journal.pgen.1003681)
Supplement: Table S4 — Gender-specific association analysis at 8 associated loci. (DOC) [file pgen.1003681.s005.doc]

**Supplementary Table S4 Gender-specific association analysis at 8 associated loci**

| **Information** |  |  |  |  |  | **Discovery** | |  | **Follow-up** | |  | **Combined** | |  |  | | | | |
| --- | --- | --- | --- | --- | --- | --- | --- | --- | --- | --- | --- | --- | --- | --- | --- | --- | --- | --- | --- |
| **Trait** | **SNP** | **Gene** | **All^1^** | **EAF^2^** | **Sex** | **beta** | **P-val** | **n** | **beta** | **P-val^3^** | **n** | **beta** | **P-val** | ***P_2GC_^4^*** | **p-value for Sex-difference** | |  | | |
| **SNPs from genome-wide association Analysis** | | | |  |  |  |  |  |  |  |  |  |  |  |  | |  |  |  |
| WC_BMI_pooled | rs2075064 | *LHX2* | t/c | 0.13 | M | -0.099 | 7.69E-04 | 5967 | -0.062 | 5.05E-02 | 3149 | -0.085 | 2.49E-04 | 3.51E-04 | 0.46 | |  | | |
|  |  |  |  |  | F | -0.074 | 1.52E-05 | 17490 | -0.035 | 1.23E-01 | 5218 | -0.065 | 1.41E-05 | 2.37E-05 |  | |  | | |
| WHR_BMI_pooled | rs6931262 | *RREB1* | t/c | 0.25 | M | 0.071 | 4.77E-03 | 4413 | 0.032 | 1.85E-01 | 2238 | 0.058 | 4.74E-03 | 5.62E-03 | 0.95 | |  | | |
|  |  |  |  |  | F | 0.061 | 8.36E-06 | 15228 | 0.038 | 7.62E-02 | 3698 | 0.056 | 3.85E-06 | 6.04E-06 |  | |  | | |
| **SNPs from cross-population interrogation** | | |  |  |  |  |  |  |  |  |  |  |  |  |  | |  |  |  |
| WHR_BMI_pooled | rs10923714 | *TBX15-WARS2* | a/g | 0.29 | M | 0.048 | 4.10E-02 | 4417 | 0.053 | 4.83E-02 | 2237 | 0.050 | 8.50E-03 | 9.84E-03 | 0.38 | |  | | |
|  |  |  |  |  | F | 0.038 | 3.25E-03 | 15215 | 0.012 | 2.87E-01 | 5195 | 0.031 | 4.69E-03 | 5.50E-03 |  | |  | | |
| WHR_BMI_pooled | rs13389219 | *GRB14* | t/c | 0.71 | M | -0.002 | 9.52E-01 | 4037 | 0.003 | 5.35E-01 | 2239 | 0.000 | 9.96E-01 | 9.96E-01 | 0.02 | |  | | |
|  |  |  |  |  | F | -0.060 | 7.14E-06 | 14713 | -0.032 | 7.75E-02 | 5187 | -0.053 | 4.43E-06 | 7.44E-06 |  | |  | | |
| WHR_BMI_pooled | rs13060013 | *ADAMTS9* | a/c | 0.22 | M | -0.001 | 9.57E-01 | 4423 | 0.072 | 9.76E-01 | 2239 | 0.023 | 2.71E-01 | 2.80E-01 | 0.27 | |  | | |
|  |  |  |  |  | F | 0.066 | 3.17E-06 | 15243 | 0.003 | 4.46E-01 | 5196 | 0.050 | 4.70E-05 | 6.45E-05 |  | |  | | |
| WHR_BMI_pooled | rs1294410 | *LY86* | t/c | 0.23 | M | -0.058 | 2.57E-02 | 4355 | -0.015 | 3.37E-01 | 2236 | -0.044 | 3.99E-02 | 4.34E-02 | 0.73 | |  | | |
|  |  |  |  |  | F | -0.059 | 1.76E-05 | 15070 | -0.029 | 1.09E-01 | 5176 | -0.052 | 1.49E-05 | 2.39E-05 |  | |  | | |
| WHR_BMI_pooled | rs1936806 | *RSPO3* | t/c | 0.29 | M | 0.042 | 8.47E-02 | 4344 | -0.004 | 5.51E-01 | 2239 | 0.026 | 1.89E-01 | 1.97E-01 | 0.67 | |  | | |
|  |  |  |  |  | F | 0.046 | 4.92E-04 | 15037 | 0.005 | 4.04E-01 | 5204 | 0.035 | 1.86E-03 | 2.36E-03 |  | |  | | |
| WHR_BMI_pooled | rs11048510 | *ITPR2-SSPN* | c/g | 0.23 | M | 0.087 | 1.07E-03 | 4406 | 0.017 | 3.27E-01 | 2239 | 0.063 | 3.58E-03 | 4.18E-03 | 0.37 | |  | | |
|  |  |  |  |  | F | 0.033 | 2.61E-02 | 15223 | 0.062 | 5.04E-03 | 5197 | 0.041 | 1.19E-03 | 1.48E-03 |  | |  | | |
| ^1^ effect allele/other allele. ^2^ effect allele frequency. ^3^ one-side test p-value. ^4^ P_2GC_: double GC-corrected p-value | | | | | | | | | | | | | | |  |  | | | |
