# Supplementary material for: Genome-Wide Association of Body Fat Distribution in African Ancestry Populations Suggests New Loci
Source: PLoS Genet. 2013 Aug 15;9(8):e1003681. doi: 10.1371/journal.pgen.1003681 (PMC3744443; doi:10.1371/journal.pgen.1003681)
Supplement: Table S5 — Local ancestry analysis for two top variants (rs2075064 at LHX2 and rs6931262 at RREB1) with single GC-corrected p-value<5×10^−8^. (DOC) [file pgen.1003681.s006.doc]

**Supplementary Table S5**. Local Ancestry Analysis for variants with Genome-wide signals for rs2075064 at *LHX2* and rs6931262 at *RREB1*.

| **Tratis** | **Loci** | **SNP** | **All^2^** | **beta** | **StdErr** | **pvalue** | **Local Ancestry SNP** |
| --- | --- | --- | --- | --- | --- | --- | --- |
| *Analysis With Adjusting for Local Ancestry* | | |  |  |  |  |  |
| WC_BMI | *LHX2* | rs2075064 | t/c | -0.073 | 0.018 | 5.84E-05 | rs10760307 |
| WHR_BMI | *RREB1* | rs6931262 | t/c | 0.060 | 0.015 | 5.98E-05 | rs2714348 |
| *Analysis Without Adjusting for Local Ancestry* | | | |  |  |  |  |
| WC_BMI | *LHX2* | rs2075064 | t/c | -0.084 | 0.018 | 2.85E-06 | N/A |
| WHR_BMI | *RREB1* | rs6931262 | t/c | 0.055 | 0.015 | 2.15E-04 | N/A |
|  |  |  |  |  |  |  |  |
| ^1^ Using CARe cohorts (ARIC, CARDIA, JHS, MESA) and WHI sample | | | | | | |  |
| ^2^ Coded/non-coded allele | |  |  |  |  |  |  |
