# Supplementary material for: Genome-Wide Association of Body Fat Distribution in African Ancestry Populations Suggests New Loci
Source: PLoS Genet. 2013 Aug 15;9(8):e1003681. doi: 10.1371/journal.pgen.1003681 (PMC3744443; doi:10.1371/journal.pgen.1003681)
Supplement: Text S1 — Study specific methods. (DOC) [file pgen.1003681.s007.doc]

**Text S1**

**Study Specific Methods**

*Discovery*

CARE Cohorts

**ARIC Study** The ARIC study is a multi-center prospective investigation of atherosclerotic disease in a bi-racial population [1]. Briefly, White and African American men and women aged 45-64 years at baseline were recruited from 4 communities: Forsyth County, North Carolina; Jackson, Mississippi; suburban areas of Minneapolis, Minnesota; and Washington County, Maryland. A total of 15,792 individuals participated in the baseline examination in 1987-1989, with follow-up examinations in approximate 3-year intervals, during 1990-1992, 1993-1995, and 1996-1998. The present study consists of African ancestry participants only. This study was approved by the institutional review board at each field center, and this analysis was approved by the University of North Carolina at Chapel Hill School of Public Health institutional review board on research involving human subjects. All subjects provided written informed consent.

Anthropometric measurements were performed were made with the participants wearing light-weight, non-constricting underwear and no shoes. Weight was measured at all clinic visits to the nearest pound, and height was measured without shoes to the nearest centimeter. Body Mass Index (BMI) was computed as weight (in kg) divided by height­­­squared (in meters). Waist circumference (WC) was measured at the level of the umbilicus at the end of an exhalation to the nearest centimeter; hip girth was measured at the level of maximal protrusion of the gluteal muscles (hips). Both waist and hip circumferences were measured to the nearest centimeter, rounding down. Waist-hip ratio (WHR) was computed as waist circumference (cm) divided by hip circumference (cm). Data from the first visit was used for all analyses.

**CARDIA**: The Coronary Artery Risk Development In Young Adults (CARDIA) study recruited a cohort of young white and black adults, ranging from 18-30 years at baseline [2]. Participants were recruited from four sites between 1985-1986: Birmingham, AL, Chicago, IL, Minneapolis, MN, and Oakland, Ca; the present study consists of African ancestry participants only. Follow-up visits occurred at Years 2, 5, 7, 10, 15, and 20. BMI and waist were determined at all exams (0,2,5,7,10,15,20,25 years after baseline). Hip circumference was measured at all exams except for 15 and 20. Height was measured in cm, rounded to the nearest 1/2 cm, and body weight in lbs, rounded to the nearest 1/2 lb, using the Detecto Model #68965 scale. Waist and hip girth were measured using a Gulick II Plus 300 cm anthropometric tape to the nearest 1/2 cm. Waist girth was obtained with the tape horizontally at a level laterally that is midway between the iliac crest and the lowest lateral portion of the rib cage and anteriorly midway between the xiphoid process of the sternum and the umbilicus; hip girth was obtained at the level of the symphysis pubis anteriorly and posteriorly at the level of the maximal protrusion of the gluteal muscles. The study was approved by the institutional review board at University of Alabama at Birmingham.

**Cleveland Family Study** (CFS): CFS is a family-based longitudinal cohort study designed to study the genetic basis of obstructive sleep apnea [3]. Index probands with a laboratory confirmed diagnosis of obstructive sleep apnea, and at least two first-degree relatives available to be studied were recruited along with family members from the Cleveland metropolitan area. Initially, neighborhood controls and their relatives were also recruited. Participants were studied at up to four exams each in approximate 4-year intervals. Only individuals over age 18 were included in this analysis. The study was approved by the institutional review board at University Hospitals Case Medical Center. All participants provided written informed consent.

Anthropomorphic measurements were performed with the participants wearing light-weight clothing including non-constrictive underwear and without shoes. Height was measured to the nearest 0.1cm and weight was measured to the nearest 0.1 kg with a digital scale. BMI was computed as the ratio of weight to height squared. Waist circumference was measured at the narrowest point of the torso between the rib cage and the iliac crest to the nearest 0.1 cm. Hip circumference was measured at the level of maximal protrusion of the gluteal muscles to the nearest 0.1 cm. All measurements were made in duplicate and averaged. Data from the last available exam was used for all analyses.

**JHS**: The Jackson Heart Study is a longitudinal population-based study from Jackson MS. Ovearll,the sample is comprised 5,302 African-American men and women identified to participate between 2000 and 2004 (visit 1) from the tricounty area that encompasses Jackson MS [4]. Anthropometry was performed with the participant wearing an examination gown. Body weight was measured using a digital scale (Detector, Model #437) and height was measured using a wall-mounted stadiometer with the participant standing erect on the horizontal platform with her/his head, shoulders, hips, and heels against the wall. Body mass index (BMI) was calculated as weight (in kilograms) divided by the square of height (in meters). Two measures of waist (horizontally at the level of the umbilicus, in the standing position) and hip (at the level of the widest circumference over the greater trochanter) were averaged to determine baseline WC and hip circumference, respectively, for each participant. Both circumferences were measured at the end of a gentle expiration while the participant was standing. The study was approved by the institutional review board at Jackson State University, Tougaloo College and the University of Mississippi Medical Center.

**MESA** MESA is a prospective, population-based multicenter cohort study designed to investigate the characteristics of subclinical atherosclerosis and the risk factors for progression of subclinical disease to clinical cardiovascular disease (CVD) among multiple ethnic groups. The study design, detailing the recruitment from the population of individuals without clinical CVD at baseline has been described previously [5]. Multi-Ethnic Study of Atherosclerosis (MESA) is comprised of 6,814 adults (38% white, 28% African ancestry, 22% Hispanic, and 12% Chinese) from the following regions: Forsyth County, North Carolina, Northern Manhattan and the Bronx, New York, Baltimore City and Baltimore County, Maryland, St.Paul, Minnesota, Chicago, Illinois, and Los Angeles County, California. The institutional review board at each participating site approved these studies, and all participants provided written informed consent. Enrolled participants were aged 45 to 84 years, free of cardiovascular disease at enrollment (2000-2002), and were of African ancestry for the present study. Height, weight, waist circumference, and hip circumference were obtained at the baseline examination using standard protocols. Briefly, all participants were measured wearing light clothing and no shoes. Height was measured using Accu-Hite stadiometers to the nearest 0.1 cm. Body weight was measured to the nearest pound using a Detecto Platform Balance Scale. Girths (waist at the umbilicus and hips at the maximal circumference of buttocks) were measured in the standing position to the nearest 0.1 cm using a Gulick II 150-cm anthropometric tape steel measuring tape with standard 4 oz. tension. No measurement was taken around clothing. Waist-hip ratio (WHR) was calculated from waist and hip girth measurements. Body mass index (BMI) was defined as weight in kilograms divided by height in meters squared.

**The Healthy Aging in Neighborhoods of Diversity across the Life Span Study (HANDLS)** HANDLS is a community-based epidemiologic study examining the associations of race and socioeconomic status (SES) and age-related health disparities among African Americans and whites in Baltimore.Briefly, HANDLS is an area probability sample of Baltimore using the 2000 Census, resulting in the recruitment of 3722 participants. Anthropometric measurements were obtained at the first study visit. Body Mass Index (BMI) was defind as weight (in kg) divided by height squared (in meters). Waist circumference was measured at the umbilicus at maximal circumference from exhalation, hip circumference was measured at the maximum circumference of the gluteal muscles, both to the nearest centimeter. The study was approved by MedStar Institutional Review Board.

**Family Heart Study** The Family Heart Study (<https://dsgweb.wustl.edu/PROJECTS/MP1.html>) began in 1992, and subjects were recruited for an extensive clinical examination during the years 1994-96. A second visit, approximately 8 years later, was conducted, in which a sample of African-American families (N=624) was recruited at the University of Alabama in Birmingham field center. Anthropometric measurements were performed with the participants wearing a scrub suit or examination gown, non-constricting underwear and no shoes. Participants were given the opportunity to empty their bladder before taking measurements. Weight was measured to the nearest pound, and height was measured without shoes to the nearest centimeter. Body Mass Index (BMI) was computed as weight (in kg) divided by height­­­squared (in meters). Waist circumference (WC) was measured at the level of the umbilicus at the end of an exhalation to the nearest centimeter; hip girth was measured at the level of maximal protrusion of the gluteal muscles (hips). Both waist and hip circumferences were measured to the nearest centimeter, rounding down. Waist-hip ratio (WHR) was computed as waist circumference (cm) divided by hip circumference (cm). The study was approved by the institutional review board at University of Alabama at Birmingham and Washington University in St.

**GeneSTAR** The Genetic Study of Atherosclerosis Risk (GeneSTAR) is a prospective study of CAD, stroke, and other vascular diseases in European- and African-American families [6,7]. Probands were identified with documented coronary disease prior to age 60 in one of ten Baltimore area hospitals. Their initially healthy siblings who were younger than age 60 were recruited and screened from 1983-2006, and the offspring of either the probands or healthy siblings as well as the co-parents of the offspring who were between the ages of 21 and 80 were recruited and screened from 2003-2006. This study was approved by the Johns Hopkins Medicine Institutional Review Board, and all subjects gave written informed consent prior to participation. All anthropometric measurements were performed with the participant wearing light clothing and no shoes. Weight was measured in pounds on a calibrated balance scale, and height was measured in inches using a stadiometer. Body mass index (BMI) was calculated as weight(kg)/height(m)2. Waist circumference (WC) was measured in inches in the standing position at the level of the iliac crest, and hip circumference was measured in inches at the widest level. The average of two measurements for the waist and hips was used; a third measurement was taken if the first two measurements varied by half an inch or more. Waist to hip ratio (WHR) was calculated as waist(cm)/hip(cm). Data from the first visit was used for all analyses.

**GENOA** The GENOA study is one of the four networks in the Family Blood Pressure Program recruited hypertensive black and non-Hispanic white sibships for linkage and family-based association studies to investigate genetic contributions to blood pressure and the cardiac and renal complication of hypertension in multiple racial groups [8,9]. Participant recruitment for GENOA (Exam 1, 1995-2000 and Exam 2, 2000-2005) was population-based in 2 geographic locations: Jackson, Mississippi and Rochester, Minnesota. African Americans in the study were located solely at the GENOA Jackson field center. The study was approved by the institutional review boards of all the participating institutions (University of Michigan Health Sciences and Behavioral Sciences IRB and University of Mississippi Medical Center IRB) and participants gave written informed consent. Anthropometric measurements were performed were made with the participants wearing light-weight, non-constricting underwear and no shoes. Weight was measured at all clinic visits to the nearest pound, and height was measured without shoes to the nearest centimeter. Body Mass Index (BMI) was computed as weight (in kg) divided by height­­­squared (in meters). Waist circumference (WC) was measured at the level of the umbilicus at the end of an exhalation to the nearest centimeter; hip girth was measured at the level of maximal protrusion of the gluteal muscles (hips). Both waist and hip circumferences were measured to the nearest centimeter, rounding down. Waist-hip ratio (WHR) was computed as waist circumference (cm) divided by hip circumference (cm). Data from the first visit was used for all analyses.

**Health ABC Methods** The Health ABC study cohort consists of 3,075 well-functioning black and white men and women aged 70–79. White participants were recruited from a random sample of Medicare beneficiaries in the zip codes in and surrounding Pittsburgh, Pennsylvania, and Memphis, Tennessee. Black participants were recruited from all age-eligible residents of the areas in and surrounding Pittsburgh and Memphis. The University of Pittsburgh, University of California, San Francisco and University of Tennessee, Memphis, institutional review boards approved the study, and all participants provided written informed consent prior to participation. Of 3,075 participants at baseline, 1137 African Americans had both genotype and phenotype data available for this analysis. Anthropometric measurements were taken at each visit. Weight was measured on a standard balance scale and height was measured with a stadiometer. Body mass index was calculated as weight in kilograms divided by height in meters squared. Waist circumference was measured by trained technicians using a tape measure at the level of the umbilicus directly on the skin. Data from the baseline visit was used for all analyses. No hip measurement was collected in Health ABC.

**HUFS Methods** The Howard University Family Study (HUFS) is a population-based family study of African Americans in the Washington, D.C. metropolitan area, USA [10]. Ethical approval for the study was obtained from the Howard University Institutional Review Board (IRB). The major objective of the HUFS was enroll and examine a randomly ascertained cohort of African American families, along with a set of unrelated individuals, to study the genetic and environmental basis of common complex diseases including hypertension, obesity, and associated phenotypes. Participants were recruited through door-to-door canvassing, advertisements in local print media and at health fairs and other community gatherings. Families and unrelated individuals were not ascertained based on any phenotype. All subjects provided written informed consent. During a clinical examination, demographic information was collected by interview. Weight, height, waist circumference, and hip circumference were measured using standard methods. Weight was measured in light clothes on an electronic scale to the nearest 0.1 kg. Height was measured with a stadiometer to the nearest 0.1 cm. Body mass index (BMI) was computed as weight divided by the square of the height. Waist circumference (WC) was measured at the level of the umbilicus at the end of an exhalation. Hip circumference was measured at the level of maximal protrusion of the gluteal muscles (hips). Both waist and hip circumferences were measured to the nearest centimeter. Waist-hip ratio (WHR) was computed as waist circumference (cm) divided by hip circumference (cm).

**HYPERGen** Study participants were enrolled in the HyperGEN (Hypertensive Genetic Epidemiology Network) study. HyperGEN is part of the Family Blood Pressure Program funded by the National Heart Lung and Blood Institute and was designed to study the genetics of hypertension and related conditions Participants were recruited from multiply-affected hypertensive sibships ascertained through population-based cohorts or from the community-at-large. The study was later extended to include siblings and offspring of the original sibpair. Probands were identified by the onset of hypertension before age 60 and the presence of at least one additional hypertensive sibling who was willing to participate. Recruitment, clinical measurement, and DNA isolation were completed in 2003. Two of four centers (AL, NC) recruited 1,277 African Americans, while three centers (NC, MN, and UT) recruited Caucasians. The study was approved by the University of Alabama’s Internal Review Board for Human Use, the Washington University Human Research Production Office, the University of North Carolina’s Office of Human Research Ethics and the Medical College of Wisconsin’s Office of Human Research Protection Program. All subjects provided written informed consent. Body Mass Index (BMI) was computed as weight (in kg) divided by height­­­squared (in meters). Waist circumference (WC) and hip girth were measured to the nearest centimeter, rounding down. Waist-hip ratio (WHR) was computed as waist circumference (cm) divided by hip circumference (cm) [11].

**MESA/SHARe Family Study** Participants in the present study were selected from the MESA cohort. MESA is a prospective, population-based cohort study designed to investigate the characteristics of subclinical atherosclerosis and the risk factors for progression of subclinical disease to clinical cardiovascular disease (CVD) among multiple ethnic groups. Details of the study design and objectives have been published previously [5]. In addition, participants were also recruited as part of a MESA Family Study, which aimed to locate and identify genes contributing to the genetic risk for CVD by looking at the early changes of atherosclerosis within families (mainly siblings). MESA Family participants underwent the same examination as MESA participants during May 2004 - May 2007. In a small proportion of subjects, parents of MESA index subjects participating in MESA Family were studied but only to have blood drawn for genotyping. DNA was extracted and lymphocytes immortalized for 1633 non-classic MESA family members (950 African Americans and 683 Hispanic-Americans) from 594 families, yielding 3,026 sibpairs. The institutional review board at each participating site (Cedars-Sinai and University of Washington Institutional Review Board) approved these studies, and all participants provided written informed consent. Anthropometric measurements were obtained using standard protocols as previously described for MESA. Height and weight were measured to the nearest 0.1 cm and pound, respectively. Body mass index (BMI) was defined as weight in kilograms divided by height in meters squared and was used as a measure of overall obesity. Waist circumference (at the umbilicus) and hip circumference (at the maximal circumference of buttocks) were measured to the nearest 0.1 cm. The waist/hip ratio (WHR) and waist circumference were used as indices of body fat distribution. Enrolled participants were aged 39 to 91 years, of African American ancestry, and measurements used in this paper were obtained at the first MESA study visit.

**WHI** 8515 self identified African American and 3642 self identified Hispanic subjects from WHI, who had consented to genetic research were selected for WHI SHARe (n=12157). DNA was extracted by the Specimen Processing Laboratory at the Fred Hutchinson Cancer research Center (FHCRC) using specimens that were collected at the time of enrollment of the subjects in WHI. Weight, height, waist and hip circumference were collected using standard protocols. The study was approved by the institutional review board at Fred Hutchinson Cancer Research Center.

*Replication Cohorts*

*Genome-wide association study of breast cancer in African Americans (AABC).*

AABC consists of 9 studies [12,13]. Three of these studies had information on waist and hip measurements and contributed to the GWAS. In these studies, weight and height information was based on self-report and waist and hip circumferences were measured.

T*he Multiethnic Cohort Study (MEC):* The MEC is a prospective cohort study of 215,000 men and women in Hawaii and Los Angeles between the ages of 45 and 75 years at baseline (1993-1996) [14]. Through December, 31 2007, a nested breast cancer (BC) case-control study in the MEC included 556 African American cases (544 invasive and 12 in situ) and 1,003 African American controls. The study was approved by the institutional review board at the University of Southern California Institutional Review Board.

*The Women’s Circle of Health Study (WCHS):* The WCHS is a case-control study of breast cancer in the New York City boroughs (Manhattan, the Bronx, Brooklyn and Queens) and in seven counties in New Jersey (Bergen, Essex, Hudson, Mercer, Middlesex, Passaic, and Union) [15]. Eligible cases included women with invasive breast cancer between 20 and 74 years of age; controls were identified through RDD. The WCHS contributed 272 invasive African American cases and 240 African American controls. The study was approved by the institutional review board at Roswell Park Cancer Institute.

*The Carolina Breast Cancer Study (CBCS):* The CBCS is a population-based case-control study conducted between 1993 and 2001 in 24 counties of central and eastern North Carolina [16]. Cases were identified by rapid case ascertainment system in cooperation with the North Carolina Central Cancer Registry and controls were selected from the North Carolina Division of Motor Vehicle and United States Health Care Financing Administration beneficiary lists. Participants’ ages ranged from 20 to 74 years. For stage 1, DNA samples were provided from 656 African American cases with invasive breast cancer and 608 African American controls. The study was approved by the institutional review board at the University of North Carolina.

*Genome-wide association study of prostate cancer in African Americans (AAPC).*

AAPC includes 14 case-control studies of prostate cancer in men of African ancestry [17,18]. Two of these studies had information on waist and hip measurements and contributed to the GWAS. In these studies, weight and height information was based on self-report and waist and hip circumferences were measured.

*The Multiethnic Cohort (MEC):* The MEC is discussed above [14]. Through January 1, 2009 the African American prostate cancer (PC) case-control study in the MEC included 1,095 cases and 1,096 controls. The study was approved by the institutional review board at the University of Southern California.

*Prostate Cancer Case-Control Studies at MD Anderson (MDA):* Participants in this study were identified from epidemiological prostate cancer studies conducted at the University of Texas M.D. Anderson Cancer Center in the Houston Metropolitan area since 1996. Cases were accrued from six institutions in the Houston Medical Center and were not restricted with respect to Gleason score, stage or PSA. Controls were identified via random-digit-dialing or among hospital visitors and they were frequency matched to cases on age and race. Lifestyle, demographic, and family history data were collected using a standardized questionnaire. These studies contributed 543 African American cases and 474 controls to this study [19]. The study was approved by the institutional review board at the University of Texas MD Anderson Cancer Center.

**Black Women’s Health Study** The BWHS began in 1995 when African American women from across the U.S. enrolled by completing postal health questionnaires [20]. The cohort followed comprises 59,000 women aged 21-69 years at baseline; median age at entry was 38 years and participants were about equally from the Northeast, South, Midwest and West. Participants are followed through biennial postal questionnaires. At baseline, BWHS participants provided information on a wide range of variables, including demographic factors, use of medical care, reproductive and medical history, cigarette and alcohol use, current weight and weight at age 18, height, waist and hip circumference, medication use, and physical activity. Biennial follow-up questionnaires ascertain new cases of specific illnesses and update information on weight, physical activity, smoking, alcohol use, and other factors.

At baseline participants reported their current weight, height, weight, and waist and hip circumferences. Follow-up questionnaires have updated weight every two years and waist and hip circumferences in 2005. In 2001 we conducted a validation study of anthropometric measures among 115 BWHS participants from the Washington D.C. area. Spearman correlations for self-reported versus technician-measured weight, height, waist circumference, and hip circumference were 0.97, 0.93, 0.75, and 0.74 respectively. Body Mass Index (BMI) was computed as weight (in kg) divided by height­­­squared (in meters). Waist-hip ratio (WHR) was computed as waist circumference (cm) divided by hip circumference (cm). Baseline data were used for all analyses.

Genotyped samples are from controls in a case-control study of breast cancer nested in the BWHS. A total of 1526 samples with complete baseline data on BMI and waist and hip circumferences were genotyped for 11 SNPs. 27 individuals were excluded because they failed genotyping. The final analytic sample included samples from 1,499 African American women. Genotyping was carried out at the Broad Institute Center for Genotyping and Analysis using the Sequenom MassArray iPLEX technology. Sixty-four blinded duplicate samples were included to assess reproducibility of the genotypes. An average reproducibility of 99.6% was obtained among the blinded duplicates. All SNPs with calling rate < 90% or a deviation from Hardy-Weinberg equilibrium in the control sample at p < 0.001 were excluded. All the 11 SNPs passed quality control requirements. We genotyped the top 30 ancestral informative markers (AIMs) from the phase 3 admixture panel (PMID: 16186815) to estimate and control for population stratification due to European admixture. We have shown that ancestry estimates using this set of 30 AIMs have an excellent correlation (r=0.89) with estimates from the whole admixture panel [21].

We estimated individual admixture proportions using a Bayesian approach as implemented in the ADMIXMAP software [22]. We assessed convergence of the admixture estimates by examination of the cumulative posterior means over all the iterations. For the continuous traits WC and WHR, residuals were created adjusted for age, age squared, and region of residence. Residuals were then inverse-normally transformed. The traits WC adjusted for BMI and WHR adjusted for BMI were created by further adjusting for BMI in the residual model and then inverse-normally transforming the residuals. We used an additive linear model for associations with genotype, adjusting for percentage of European ancestry as a continuous variable. All regression models were run using the SAS statistical software version 9.1.3 (SAS Institute Inc., Cary, NC, USA). The study was approved by the institutional review board at Boston University.

**Cardiovascular Health Study** CHS is a population-based cohort study of risk factors for CHD and stroke in adults ≥65 years conducted across four field centers [23]. The original predominantly Caucasian cohort of 5,201 persons was recruited in 1989-1990 from random samples of the Medicare eligibility lists; subsequently, an additional predominantly African-American cohort of 687 persons was enrolled subsequently for a total sample of 5,888. DNA was extracted from blood samples drawn on all participants at their baseline examination in 1989-90. In 2010, genotyping was performed at the General Clinical Research Center's Phenotyping/Genotyping Laboratory at Cedars-Sinai using the Illumina HumanOmni1-Quad_v1 BeadChip system on 844 African-American CHS participants who consented to genetic testing, and had DNA available for genotyping. Genotyping was attempted in 844 participants, and was successful in 823 persons; the latter constitute the CHS sample for this study. Weight was measured at all clinic visits to the nearest pound, and height was measured to the nearest centimeter. Body Mass Index (BMI) was computed as weight (in kg) divided by height­­­ squared (in meters). Both waist (WC) and hip circumferences were measured to the nearest centimeter. Waist-hip ratio (WHR) was computed as waist circumference (cm) divided by hip circumference (cm). Data from the baseline visit was used for all analyses. The study was approved by the institutional review board at University of Washington.

**SIGNET** The Sea Islands Genetics Network (SIGNET) study consists of the REasons for Geographic And Racial Differences in Stroke (REGARDS), the Sea Islands Genetic African American Registry (Project SuGAR), a COBRE for Oral Health, and the Systemic Lupus Erythematosus in Gullah Health study (SLEIGH). All subjects are African Americans (AA), and all provided written informed consent.

REGARDS is an observational cohort of 30,239 AA and white men and women enrolled in their homes after a telephone interview in 2003-2007 [24]. Participants were a national sample oversampled from the southeastern stroke belt (56%) and were 58% female and 42% black by design. Participants were followed every 6 months by telephone to ascertain health outcomes, with validation of stroke, coronary heart disease, death and other ancillary study endpoints. GWAS genotyping was completed among 2398 AA participants including 1149 with diabetes and 1249 without diabetes. Project SuGAR patients with T2DM in AA families with multiple affected members living on the Sea Islands [25]. Inclusion criteria included at least one affected sibling pair, no more than one parent with T2DM, and at least one parent alive. All consenting members of families meeting these criteria were enrolled. Medical, anthropometric, family and medical history, physical examination and laboratory testing were obtained.  GWAS genotyping was completed in 1,176 participants; 967 with diabetes and 193 without.  The COBRE for Oral Health enrolled 226 AA persons with type 2 diabetes ≥18 years old and not edentulous [26]. Participants lived along coastal South Carolina and 30 miles inland. Subjects answered a detailed questionnaire focusing on medical and dental history and underwent an oral examination to document periodontal health. SLEIGH is a population based case-control study of risk factors for systemic lupus erythematosus (SLE). Inclusion criteria were: 1) age 2 years or older, 2) self-identification as AA Gullah from the Sea Islands of South Carolina, with no known ancestors who were not of Gullah lineage, 3) at least 4 of the 11 American College of Rheumatology classification criteria for SLE, 4) and being able to speak and understand English. First-degree relatives of SLE probands are invited to enroll. Healthy AA control subjects without evidence of autoimmune or connective tissue disease, or of family history of SLE, from the Sea Islands were age- and sex-matched to cases. Genotype data was generated in 93 SLEIGH participants, including 15 with diabetes and 77 without.

Anthropometric measurements were made with participants wearing light-weight, non-constricting underwear and no shoes. Weight was measured to the nearest pound, and height without shoes to the nearest centimeter. Body Mass Index (BMI) was computed as weight (in kg) divided by height­­­squared (in meters). Waist circumference (WC) was measured at the level of the umbilicus at the end of an exhalation to the nearest centimeter; hip girth was measured at the level of maximal protrusion of the gluteal muscles (hips). Both waist and hip circumferences were measured to the nearest centimeter, rounding down. Waist-hip ratio (WHR) was computed as waist circumference (cm) divided by hip circumference (cm).

Samples from 3571 SIGNET individuals with WC were genotyped using the Affymetrix Genome-Wide Human SNP Array 6.0, among which 1185 individuals are measured for WHR. We used the EIGENSTRAT method [27] to compute the first 10 principal components in REGARDS and non-REGARDS populations. For the continuous traits WC and WHR, sex-specific, T2D status-specific and study-specific (REGARDS and non-REGARDS) residuals were created adjusted for age and age squared. Residuals were then inverse-normally transformed. The traits WC adjusted for BMI and WHR adjusted for BMI were created by further adjusting for BMI in the residual model and then inverse-normally transforming the residuals. We used an additive linear model for associations with genotype, adjusting for principal components. For family data as in non-REGARDS population, familial correlation was adjusted using the variance component models. Regression models were run either using PLINK package for unrelated individual data or MERLIN package for family data. The REGARDS study was approved by the institutional review board at University of Virginia and University Alabama at Birmingham. The SUGAR study was approved by the institutional review board at University of Virginia and Medical University of South Carolina.

**Genetic Contributors to Diabetes**

The study was approved by the institutional review board at University of Alabama Birmingham (X100128020).

**Metabolomics Profiling Gullah**

The study was approved by the institutional review board at University of Alabama Birmingham (X070221003).

Reference List

1. 1989) The Atherosclerosis Risk in Communities (ARIC) Study: design and objectives. The ARIC investigators. Am J Epidemiol 129: 687-702.

2. Friedman GD, Cutter GR, Donahue RP, Hughes GH, Hulley SB et al. (1988) CARDIA: study design, recruitment, and some characteristics of the examined subjects. J Clin Epidemiol 41: 1105-1116.

3. Redline S, Tishler PV, Tosteson TD, Williamson J, Kump K et al. (1995) The familial aggregation of obstructive sleep apnea. Am J Respir Crit Care Med 151: 682-687.

4. Taylor HA, Jr. (2005) The Jackson Heart Study: an overview. Ethn Dis 15: S6-3.

5. Bild DE, Bluemke DA, Burke GL, Detrano R, ez Roux AV et al. (2002) Multi-ethnic study of atherosclerosis: objectives and design. Am J Epidemiol 156: 871-881.

6. Mora S, Yanek LR, Moy TF, Fallin MD, Becker LC et al. (2005) Interaction of body mass index and framingham risk score in predicting incident coronary disease in families. Circulation 111: 1871-1876.

7. Bordeaux BC, Qayyum R, Yanek LR, Vaidya D, Becker LC et al. (2010) Effect of obesity on platelet reactivity and response to low-dose aspirin. Prev Cardiol 13: 56-62.

8. Daniels PR, Kardia SL, Hanis CL, Brown CA, Hutchinson R et al. (2004) Familial aggregation of hypertension treatment and control in the Genetic Epidemiology Network of Arteriopathy (GENOA) study. Am J Med 116: 676-681.

9. 2002) Multi-center genetic study of hypertension: The Family Blood Pressure Program (FBPP). Hypertension 39: 3-9.

10. Adeyemo A, Gerry N, Chen G, Herbert A, Doumatey A et al. (2009) A genome-wide association study of hypertension and blood pressure in African Americans. PLoS Genet 5: e1000564.

11. Williams RR, Rao DC, Ellison RC, Arnett DK, Heiss G et al. (2000) NHLBI family blood pressure program: methodology and recruitment in the HyperGEN network. Hypertension genetic epidemiology network. Ann Epidemiol 10: 389-400.

12. Haiman CA, Chen GK, Vachon CM, Canzian F, Dunning A et al. (2011) A common variant at the TERT-CLPTM1L locus is associated with estrogen receptor-negative breast cancer. Nat Genet 43: 1210-1214.

13. Chen F, Chen GK, Millikan RC, John EM, Ambrosone CB et al. (2011) Fine-mapping of breast cancer susceptibility loci characterizes genetic risk in African Americans. Hum Mol Genet 20: 4491-4503.

14. Kolonel LN, Henderson BE, Hankin JH, Nomura AM, Wilkens LR et al. (2000) A multiethnic cohort in Hawaii and Los Angeles: baseline characteristics. Am J Epidemiol 151: 346-357.

15. Ambrosone CB, Ciupak GL, Bandera EV, Jandorf L, Bovbjerg DH et al. (2009) Conducting Molecular Epidemiological Research in the Age of HIPAA: A Multi-Institutional Case-Control Study of Breast Cancer in African-American and European-American Women. J Oncol 2009: 871250.

16. Newman B, Moorman PG, Millikan R, Qaqish BF, Geradts J et al. (1995) The Carolina Breast Cancer Study: integrating population-based epidemiology and molecular biology. Breast Cancer Res Treat 35: 51-60.

17. Haiman CA, Chen GK, Blot WJ, Strom SS, Berndt SI et al. (2011) Characterizing genetic risk at known prostate cancer susceptibility loci in African Americans. PLoS Genet 7: e1001387.

18. Haiman CA, Chen GK, Blot WJ, Strom SS, Berndt SI et al. (2011) Genome-wide association study of prostate cancer in men of African ancestry identifies a susceptibility locus at 17q21. Nat Genet 43: 570-573.

19. Strom SS, Gu Y, Zhang H, Troncoso P, Babaian RJ et al. (2004) Androgen receptor polymorphisms and risk of biochemical failure among prostatectomy patients. Prostate 60: 343-351.

20. Palmer JR, Wise LA, Horton NJ, ms-Campbell LL, Rosenberg L (2003) Dual effect of parity on breast cancer risk in African-American women. J Natl Cancer Inst 95: 478-483.

21. Ruiz-Narvaez EA, Rosenberg L, Wise LA, Reich D, Palmer JR (2011) Validation of a small set of ancestral informative markers for control of population admixture in African Americans. Am J Epidemiol 173: 587-592.

22. Hoggart CJ, Parra EJ, Shriver MD, Bonilla C, Kittles RA et al. (2003) Control of confounding of genetic associations in stratified populations. Am J Hum Genet 72: 1492-1504.

23. Fried LP, Borhani NO, Enright P, Furberg CD, Gardin JM et al. (1991) The Cardiovascular Health Study: design and rationale. Ann Epidemiol 1: 263-276.

24. Howard VJ, Cushman M, Pulley L, Gomez CR, Go RC et al. (2005) The reasons for geographic and racial differences in stroke study: objectives and design. Neuroepidemiology 25: 135-143.

25. Sale MM, Lu L, Spruill IJ, Fernandes JK, Lok KH et al. (2009) Genome-wide linkage scan in Gullah-speaking African American families with type 2 diabetes: the Sea Islands Genetic African American Registry (Project SuGAR). Diabetes 58: 260-267.

26. Bandyopadhyay D, Marlow NM, Fernandes JK, Leite RS (2010) Periodontal disease progression and glycaemic control among Gullah African Americans with type-2 diabetes. J Clin Periodontol 37: 501-509.

27. Price AL, Patterson NJ, Plenge RM, Weinblatt ME, Shadick NA et al. (2006) Principal components analysis corrects for stratification in genome-wide association studies. Nat Genet 38: 904-909.
